# The Etiology of Childhood Pneumonia in Mali: Findings From the Pneumonia Etiology Research for Child Health (PERCH) Study

**DOI:** 10.1097/INF.0000000000002767

**Published:** 2021-08-25

**Authors:** Milagritos D. Tapia, Mamadou Sylla, Amanda J. Driscoll, Aliou Touré, Nana Kourouma, Seydou Sissoko, Boubou Tamboura, Abdoul Aziz Diakité, Sandra Panchalingam, Adama M. Keïta, Sharon Tennant, Uma Onwuchekwa, Anna Roose, Maria Deloria Knoll, Melissa M. Higdon, Christine Prosperi, Laura L. Hammitt, Daniel R. Feikin, David R. Murdoch, Katherine L. O’Brien, Samba O. Sow, Karen L. Kotloff

**Affiliations:** From the * Department of Pediatrics, Center for Vaccine Development and Global Health, University of Maryland School of Medicine, Baltimore, Maryland; † Centre pour le Développement des Vaccins-Mali, Bamako, Mali; ‡ Department of International Health, International Vaccine Access Center, Johns Hopkins Bloomberg School of Public Health, Baltimore, Maryland; § Department of Pediatrics, Hôpital Gabriel Touré, Bamako, Mali; ¶ Department of Medicine, Center for Vaccine Development and Global Health, University of Maryland School of Medicine, Baltimore, Maryland; ‖ Department of Pathology, University of Otago, Christchurch, New Zealand; ** Microbiology Unit, Canterbury Health Laboratories, Christchurch, New Zealand.

**Keywords:** pneumonia, mortality, low-income countries, Mali, etiology

## Abstract

We present findings from the Pneumonia Etiology Research for Child Health (PERCH) site in Bamako, Mali.

**Methods:**

Cases were patients 28 days to 59 months of age, admitted to hospital with severe or very severe pneumonia (2005 World Health Organization definition). Community controls were frequency matched by age. Both provided nasopharyngeal and oropharyngeal swabs for multiplex polymerase chain reaction and *Streptococcus pneumoniae* culture. Cases underwent blood culture and induced sputum culture for *Mycobacterium tuberculosis*. A subset had pleural fluid and lung aspirates collected for culture and polymerase chain reaction. Primary analyses included participants with negative or unknown HIV status (HIV−) and cases with abnormal chest radiographs (CXR+). Cases and controls were compared using logistic regression adjusting for age. Etiologic fractions were calculated by a Bayesian nested partially latent class analysis, the PERCH integrated analysis.

**Results:**

Between January 1, 2012, and January 14, 2014, we enrolled 241 CXR+/HIV− cases and 725 HIV− controls. Compared with controls, cases were more likely to have moderate-to-severe wasting (43.1% vs. 14.1%, *P* < 0.001) and stunting (26.6% vs. 9.4%, *P* < 0.001). Predominant etiologies were respiratory syncytial virus [24.0%; 95% credible interval (CrI): 18.3%–31.1%], *S. pneumoniae* (15.2%; 95% CrI: 9.5–21.6), human metapneumovirus (11.8%; 95% CrI: 8.3%–16.2%) and parainfluenza virus type 3 (9.0%; 95% CrI: 5.8%–13.3%). Case fatality was 13.3%, with *Staphylococcus aureus*, *Pneumocystis jirovecii* and *Haemophilus influenzae* type b predominating (40% of fatal cases).

**Conclusions:**

PERCH uncovered high case fatality among children with severe pneumonia in Mali, highlighting a role for new interventions (eg, respiratory syncytial virus vaccines) and a need to improve vaccine coverage and strengthen healthcare delivery.

## Abstract 2

Supplemental Digital Content is available in the text.

Pneumonia is the leading cause of postneonatal under-5 mortality worldwide with most deaths occurring in sub-Saharan Africa.^1^ Despite substantial reductions in pneumonia mortality during the Millennium Development Goal period (2000–2015), nearly 1 million children died from pneumonia in 2015.^1,2^ The aim of the multisite Pneumonia Etiology Research for Child Health (PERCH) study was to describe the etiology of severe and very severe pneumonia in hospitalized children living in 7 low- and middle-income countries in Asia and Africa to inform current and future public health priorities.^3^ One of the research sites was the Centre pour le Développement des Vaccins-Mali. Mali is a low-income, high mortality West African country with an HIV prevalence of 1.7% among women of reproductive age in Bamako.^4^ According to United Nations Children’s Fund, Mali had the eighth highest under-5 mortality rate globally at the start of PERCH in 2012, with 128 deaths occurring for every 1000 live births (90% uncertainty interval: 69–176).^5^ Before PERCH, data on the burden and etiology of pneumonia in Malian children were limited. A retrospective study of pediatric inpatients in 2002–2003 attributed 18% of admissions and 12% of deaths to pneumonia.^6^ Vaccine-preventable pneumonias [ie, those caused by *Streptococcus pneumoniae*, *Haemophilus influenzae* type b (Hib) and influenza virus] were estimated to account for at least one-third of severe episodes and two-thirds of deaths in the World Health Organization (WHO) African region in 2010.^7^ Hib conjugate vaccine was introduced into Mali’s Expanded Programme on Immunization in 2005, and 13-valent pneumococcal conjugate vaccine (PCV-13) was introduced in 2011 just before the start of PERCH enrollment. While Hib vaccine had been shown to significantly reduce rates of confirmed Hib hospitalizations in Mali,^8^ the effect of these vaccines on the burden of pneumonia in this setting was undocumented. Moreover, the burden of viral etiologies of pneumonia had not previously been systematically assessed in Mali. To address these knowledge gaps, we present findings from PERCH on the etiology and epidemiology of severe and very severe pneumonia in Malian children.

## MATERIALS AND METHODS

### Selection of Participants

#### Cases

Between January 1, 2012, and January 14, 2014, we aimed to enroll 700 cases from l’Hôpital Gabriel Touré (HGT), the primary pediatric hospital in the capital city of Bamako. Patients who were 28 days to 59 months of age, resided in Bamako, and presented to the Emergency Department or Outpatient Department with cough or difficulty breathing were screened for eligibility by trained study personnel (additional detail provided in Table, Supplemental Digital Content 1, http://links.lww.com/INF/E11).^9^ Eligible cases were those who were admitted to HGT with WHO-defined severe or very severe pneumonia (2005 definition).^10^ Severe pneumonia was defined as cough or difficulty breathing plus lower chest indrawing; very severe pneumonia was defined as cough or difficulty breathing with one or more of the following: central cyanosis, difficulty breast-feeding or drinking, vomiting everything, convulsions, lethargy, unconsciousness or head nodding. Intravenous hydration and antibiotics were provided to cases free of charge as prescribed. Oxygen was present in the intensive care unit where cases were hospitalized, but its use was inconsistent due to limited availability of the equipment required to properly administer it. Ventilators were not available during the study period.

#### Controls

A minimum of 25 controls were randomly selected from the community each month, with additional controls enrolled as needed to match surges in case enrollment during the previous month. Community controls were frequency matched to cases by age and were proportionally enrolled from quartiers (neighborhoods) in Bamako according to the residential patterns of pneumonia hospitalizations at HGT for the previous 5 years. Quartier populations based on adjusted estimates from the 2009 census were provided by Institut National de la Statistique.

### Demographic, Environmental and Clinical Data Collection

Demographic data, environmental characteristics and clinical histories were collected from cases and controls. Vaccination status was ascertained from the vaccine card or by maternal recall if no vaccination card was available. Additional clinical assessments, including temperature, pulse oximetry, respiratory rate and assessments for signs of severe disease were performed on cases at enrollment and at 24 and 48 hours after admission using standardized methods.^9^ Cases had a final vital status assessment 30 days after admission (allowable range: 21–90 days).

### Sample Collection and Laboratory Testing

As previously described,^9,11^ all cases and controls provided nasopharyngeal (NP) and oropharyngeal (OP) swabs for detection of respiratory pathogens by multiplex quantitative polymerase chain reaction (PCR; FTD Resp-33 kit; Fast-track Diagnostics, Sliema, Malta) and for culture and serotyping of *S. pneumoniae*, as well as blood for *S. pneumoniae* PCR and serum for antibiotic activity testing. Additional assays performed only for cases included blood culture and induced sputum for *Mycobacterium tuberculosis* (MTB) culture. When induced sputum could not be collected, a gastric aspirate was attempted. Pleural fluid was collected when clinically indicated, and lung aspirates were collected from a subset of clinically stable cases with dense peripheral consolidation identified on chest radiograph (CXR). Pleural fluid and lung aspirate specimens were tested by culture and, when sufficient volume was available, by PCR. When possible, study specimens from cases were collected before antibiotic administration. CXRs were obtained from each child within 72 hours of admission and interpreted by a panel of radiologists and pediatricians who had been trained in the standardized interpretation of pediatric CXRs using the WHO CXR reading framework.^12,13^ Vaccine-preventable diseases were defined as those caused by *S. pneumoniae* PCV-13 type, Hib and *Bordetella pertussis*. Data were collected on maternal HIV status during pregnancy, and participants underwent HIV testing if their parent/guardian signed a separate consent. HIV testing and classification methods are described in detail in Table, Supplemental Digital Content 1, http://links.lww.com/INF/E11.

### Data Management and Statistical Analysis

Data were entered into an electronic data capture system (The Emmes Corporation, Rockville, MD).^14^ Cases and controls were classified as HIV infected (HIV+) if they had laboratory-confirmed HIV infection. The primary analysis was restricted to HIV-uninfected (HIV−) participants, defined as those who tested negative (HIV test negative) or had unknown HIV status. Cases included in the primary analyses were those who were HIV− and had an abnormal CXR (CXR+), defined as having either alveolar consolidation, other infiltrate or both. CXR+ cases were selected as the primary analytic group because they were most likely to have an infection in their lungs. Odds ratios (ORs) and 95% confidence intervals (CIs) comparing cases and control characteristics were calculated using logistic regression and adjusting for age. ORs comparing the prevalence of organisms detected by PCR of case and control NP/OP swabs were adjusted for age and for the presence of all other pathogens detected in the specimen, generating ORs that quantify the degree of association between pneumonia and a particular pathogen among persons who are otherwise similar with respect to the other measured pathogens. Previously defined NP/OP PCR density thresholds were used to determine positivity for pathogens commonly detected in controls (*S. pneumoniae, H. influenzae*, and *Pneumocystis jirovecii*); standard assay thresholds were used for the other microorganisms included in the multiplex PCR assay.^15–17^ Secondary analyses compared the clinical characteristics and case fatality ratio (CFR) of CXR+ cases to all cases and of HIV+ cases to HIV− cases.

The percentage of pneumonia due to each pathogen was estimated using the PERCH integrated analysis (PIA) method, which is described in detail elsewhere.^3,18–20^ In brief, the PIA is a Bayesian nested partially latent class analysis that integrates the results for each case from blood culture, NP/OP PCR, whole blood PCR for pneumococcus and induced sputum culture (or gastric aspirate culture when induced sputum was not collected) for MTB. The PIA also integrates test results from controls to account for imperfect test specificity of NP/OP PCR and whole blood PCR. Blood culture results (excluding contaminants), MTB results and results from lung aspirate and pleural fluid specimens were assumed to be 100% specific (ie, the etiology for a case was attributed 100% to the pathogen that was detected in their blood by culture). To exclude nosocomial infections, lung aspirate and pleural fluid results were only included in analyses when the specimens were collected within 3 days of admission. Given the small number of children with pleural effusion confirmed on CXR, pleural fluid results were used to inform the case’s individual etiology but were not extrapolated to other cases.

The PIA estimated both the individual- and population-level etiology probability distributions, each summing to 100% across pathogens where each pathogen has a probability ranging from 0% to 100%. The population-level etiologic fraction estimate for each pathogen was approximately the average of the individual case probabilities and was provided with a 95% credible interval (95% CrI), the Bayesian analogue of the CI. The PIA model assumes that a pneumonia event is caused by a single pathogen. For cases with multiple pathogens detected in measurements assumed to be 100% specific (ie, blood cultures, lung aspirates or pleural fluid), the model cannot distinguish which pathogen is the dominant cause and distributes the etiology probability equally across the pathogens detected so that the probabilities sum to 100%. This distribution leads to an underestimation of the true cause of the disease and an overestimation of the other. For cases who are negative by one of the measurements with 100% specificity, the etiology probabilities are distributed across all potential pathogens, informed by the pathogen results for that individual, the overall case prevalence for the pathogen and its association with case status and a priori assumptions regarding test sensitivity and etiology distributions. To calculate the CFR of respiratory syncytial virus (RSV), the proportion of children who died among all those assigned to RSV is calculated at each iteration of the analysis, and those CFRs are averaged across all iterations to obtain an overall CFR, and the distribution of the iteration-specific CFRs is used to calculate a 95% CrI. This is similarly done for all the remaining (non-RSV) cases at each iteration to obtain a non-RSV CFR. Additional details about the PIA methods are presented in Table, Supplemental Digital Content 1, http://links.lww.com/INF/E11.

### Ethics

We obtained written informed consent for study participation from parents or guardians of cases and controls before performance of any study procedure. The study was approved by the ethics committee at the Faculté de Médecine, Pharmacie et Odontostomatologie in Mali and the Institutional Review Boards of the University of Maryland School of Medicine and Johns Hopkins Bloomberg School of Public Health.

## RESULTS

### Screening and Enrollment of Cases and Controls

**FIGURE 1.**
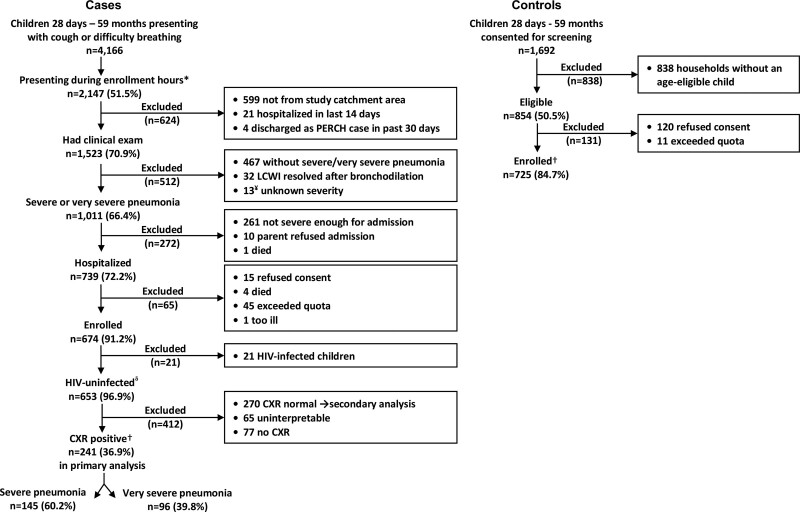
Case and control enrollment flow diagram. *Cases were systematically sampled during prespecified morning and evening shifts, and a maximum of 4–6 children were enrolled per shift. ¥Thirteen children had lower chest wall indrawing and wheeze and underwent a bronchodilator challenge; whether lower chest wall indrawing persisted was not recorded because quota had been met. Therefore, it is unknown whether these children met the criteria for severe/very severe pneumonia. δSecondary analysis includes 653 HIV-uninfected children and compares those with positive and negative CXR. †Primary analysis includes 241 children without known HIV infection [includes 164 (68%) whose HIV test was negative, and 77 (32%) whose HIV test was unknown] and had CXRs that met the WHO case definition of consolidation and/or other infiltrate along with 725 frequency-matched controls. ‡Includes 285 (39%) whose HIV test was negative, and 440 (61%) whose HIV test was unknown.

Between January 1, 2012, and January 14, 2014, 4166 children 28 days to 59 months of age visited HGT with cough and/or difficulty of breathing (Fig. 1); 2147 (51.5%) presented during enrollment hours of whom 1523 (70.9%) were screened for eligibility. Of the 1011 (66.4%) who met case eligibility criteria for severe or very severe pneumonia, 739 (72.2%) were hospitalized, 674 (91.2%) of whom were enrolled. Of the 674 enrolled cases, 470 (69.7%) consented to HIV testing and 21 (4.5%) of those tested were HIV+. Of the 653 cases with unknown HIV status or negative HIV test results, 241 (36.9%) were CXR+ and were included in the primary analysis. The majority [157/241 (65.1%)] of cases included in the primary analysis were between 28 days and 11 months of age. Among the 1692 community controls who were screened, 854 (50.5%) were eligible, of whom 725 (84.7%) were enrolled and 435 (60%) were 28 days to 11 months of age. Among the 725 enrolled controls, 285 (39.3%) consented to HIV testing, all of whom tested negative. Among the mothers who reported knowing their HIV status during pregnancy, 4 of 83 (4.8%) mothers of cases and 1 of 82 (0.1%) mothers of controls reported being HIV+. All participants with unknown HIV status [77/241 (32.0%) of cases and 440/725 (60.7%) of controls] were classified as HIV− in the primary analyses. Samples were collected as indicated in Table, Supplemental Digital Content 2, http://links.lww.com/INF/E12.

### Demographic and Clinical Features of Cases (CXR+, HIV−) and Controls

Compared with controls, cases were younger (median age: 8 vs. 9 months, *P* = 0.008) and more likely to be male (59.8% vs. 49.5%, *P* = 0.009). Cases had worse nutritional status than controls, with increased moderate and severe wasting [weight for height Z score (WHZ) <−2, 43.1% vs. 14.1%, *P* < 0.001] and stunting [height for age Z score (HAZ) <−2, 26.6% vs. 9.4%, *P* < 0.001], and were more likely to have been exposed to antibiotics (53.5% vs. 2.7%, *P* < 0.001) (Table 1). The nutritional status of cases with unknown HIV status was similar to that of cases who were HIV test negative (Table, Supplemental Digital Content 3, http://links.lww.com/INF/E13). Cases were less likely than controls to have ever been breast-fed (93.6% vs. 99.6%, *P* < 0.001) and were breast-fed for a shorter duration (median: 6.5 vs. 9 months, *P* < 0.001). There was no difference in the proportion of cases who were ever breast-fed when comparing those born to mothers who had unknown HIV status or were HIV infected during pregnancy to those born to mothers who were HIV uninfected during pregnancy [152/160 (95.0%) vs. 69/76 (90.8%), *P* = 0.215]. Vaccination status was available from vaccine cards for 62 of 241 (25.7%) of cases and 460 of 725 (63.5%) of controls, the remainder were from parental recall. There was no difference in pentavalent vaccine coverage by case-control status for participants <12 months of age (72.7% of cases and 79.5% of controls fully vaccinated for age, *P* = 0.070), but among those 12 months or older, cases were less likely to be fully vaccinated than controls (85.2% vs. 95.9%, *P* = 0.002). There were no differences in PCV-13 coverage by case-control status (Table 1).

**TABLE 1.** Demographic and Clinical Characteristics of HIV-Uninfected Cases and Controls

	All Cases(N = 653)	CXR+ Cases*(N = 241)	Controls(N = 725)	CXR+ Cases vs. Controls
	n (%)	n (%)	n (%)	
Age				*P* value
Median age in months (IQR)	6 (3–13)	8 (3–13)	9 (4–20)	0.008†
28 days to 5 months	299 (45.8)	98 (40.7)	247 (34.1)	0.002
6–11 months	148 (22.7)	59 (24.5)	188 (25.9)	0.001
12–23 months	137 (21.0)	61 (25.3)	165 (22.8)	0.035
24–59 months	69 (10.6)	23 (9.5)	125 (17.2)	Ref‡
				Adjusted *P* value§
Female	289 (44.3)	97 (40.2)	366 (50.5)	0.009
HIV exposure/infection status¶				
HIV unexposed/uninfected	442 (67.7)	158 (65.6)	283 (39)	Ref
HIV exposed/uninfected	7 (1.1)	6 (2.5)	2 (0.3)	0.078
Unknown exposure/infection status	204 (31.2)	77 (32.0)	440 (60.7)	<0.001
Respiratory tract illness (controls only)‖	N/A	N/A	299 (41.2)	
Number pentavalent vaccine doses received				
0	80 (12.5)	29 (12.3)	51 (7.0)	0.031
1	127 (19.8)	43 (18.3)	76 (10.5)	0.020
2	104 (16.2)	32 (13.6)	77 (10.6)	0.231
3+	330 (51.5)	131 (55.7)	521 (71.9)	Ref
Pentavalent: fully vaccinated for age**				
<1 year old	323 (73.4)	112 (72.7)	346 (79.5)	0.070
≥1 year old	176 (87.6)	69 (85.2)	278 (95.9)	0.002
Number of PCV doses received				
0	139 (21.7)	53 (22.6)	212 (29.3)	0.779
1	120 (18.7)	40 (17.0)	73 (10.1)	0.140
2	103 (16.1)	31 (13.2)	73 (10.1)	0.553
3+	279 (43.5)	111 (47.2)	365 (50.5)	Ref
PCV fully vaccinated for age**				
<1 year old	323 (73.4)	112 (72.7)	332 (76.5)	0.587
≥1 year old	124 (61.7)	48 (59.3)	134 (46.4)	0.397
≥1 measles vaccine dose (children > 10 months)	190 (78.5)	78 (78.0)	326 (88.1)	0.554
Breast-feeding				
Median number of months breast-fed (IQR)††	6 (2–12)	6.5 (2–12)	9 (4–17)	<0.001†
Median number of months exclusively breast-fed (IQR)††	0 (0–3)	0 (0–3)	0 (0–3)	0.646†
Ever breast-fed	607 (95.0)	221 (93.6)	720 (99.6)	<0.001
Midupper arm circumference <11.5 cm (children ≥6 months)	69 (19.5)	36 (25.2)	1 (0.2)	<0.001
WAZ by WHO standards				
≥−2	390 (59.7)	127 (52.7)	629 (87.2)	Ref
3 to < −2	123 (18.8)	43 (17.8)	65 (9.0)	<0.001
<−3	140 (21.4)	71 (29.5)	27 (3.7)	<0.001
WHZ by WHO standards				
≥−2	401 (61.5)	137 (56.9)	617 (85.9)	Ref
−3 ≤ to < −2	115 (17.6)	41 (17.0)	65 (9.1)	<0.001
<−3	136 (20.9)	63 (26.1)	36 (5.0)	<0.001
HAZ by WHO standards				
≥−2	513 (78.7)	177 (73.4)	653 (90.6)	Ref
−3 ≤ z to <−2	75 (11.5)	34 (14.1)	52 (7.2)	<0.001
<−3	64 (9.8)	30 (12.5)	16 (2.2)	<0.001
Prior antibiotic use				
Serum antibiotic activity	127 (19.5)	41 (17.1)	19 (2.7)	<0.001
Antibiotics administered at study hospital before specimen collection	29 (4.4)	7 (2.9)	N/A	
Documented antibiotic pretreatment before specimen collection‡‡	172 (26.3)	54 (22.4)	19 (2.6)	<0.001
Parental report of antibiotics	326 (57.4)	117 (54.4)	0 (0.0)	0.993
Any of above	371 (60.8)	129 (53.5)	19 (2.7)	<0.001

*CXR+ cases: finding of alveolar consolidation, other infiltrate or both on chest radiograph.

†*P* value for difference in medians calculated using Kruskal-Wallis test.

‡Reference category.

§*P* value adjusting for age in months.

¶Positive enzyme-linked immunosorbent assay (ELISA) test or reported maternal history of HIV+ during pregnancy if child is <7 months without a negative ELISA result or reported maternal history of HIV+ during pregnancy if child is ≥7 months regardless of ELISA results.

‖Respiratory tract illness defined as (1) cough and/or runny nose or (2) at least 1 of ear discharge, wheezing or difficulty breathing in the presence of either a temperature of ≥38.0°C within past 48 hours or a history of sore throat.

**For children <1 year, defined as received at least 1 dose and up-to-date for age based on the child’s age at enrolment, doses received and country schedule (allowing 4-week window each for dose). For children ≥1 year, defined as 3+ doses.

††Among those who were ever breast-fed.

‡‡Presence of antibiotics by serum, antibiotics given at the referral hospital, clinician report of antibiotics before any specimen collection or antibiotics before any specimen collection based on time of collection of that specimen and time of antibiotic administration. Only criterion applicable to controls is serum.

IQR indicates interquartile range; N/A, not applicable.

### Demographic and Clinical Features and Mortality of Severe and Very Severe Cases (CXR+, HIV−)

More than one-third [96/241 (39.8%)] of enrolled cases met the definition of very severe pneumonia. Cases with very severe pneumonia were younger than those with severe pneumonia (median age: 9 vs. 6 months, *P* < 0.001) (Table 2). Very severe cases were more likely than severe cases to present with hypoxemia (64.6% vs. 49.0%, *P* = 0.039) and grunting (72.9% vs. 56.6%, *P* = 0.005) and to have antibiotic activity detected in serum (24.2% vs. 12.4%, *P* = 0.024). Among the 233 cases with 30-day vital status available, 31 (13.3%) died in hospital or within 30 days of hospital admission. Most deaths (25/31, 80.6%) occurred in the hospital, with 5 (20%) taking place within 48 hours of admission. Compared with severe cases, very severe cases were more likely to die during their hospitalization (17.4% vs. 6.4%, *P* = 0.008) and within 30 days of hospital admission (18.5% vs. 9.9%, *P* = 0.060).

**TABLE 2.** Demographic and Clinical Characteristics of HIV-Negative CXR+ Cases, by Severity

	Severe(n = 145)	Very Severe(n = 96)	Severe vs. Very Severe
	Median (IQR)	Median (IQR)	*P* Value*
Age in months (median and IQR)	9 (4–16)	6 (2.5–12)	<0.001
	n (%)	n (%)	Age-adjusted *P* value†
28 days to 5 months	51 (35.2)	47 (49.0)	0.994
6–11 months	35 (24.1)	24 (25.0)	
12–23 months	40 (27.6)	21 (21.9)	
24–59 months	19 (13.1)	4 (4.2)	
Female	59 (40.7)	38 (39.6)	0.921
CXR result			
Any consolidation	88 (60.7)	48 (50.0)	0.127
Other infiltrate only	57 (39.3)	48 (50.0)	
Pentavalent doses received			
At least 1 dose of pentavalent	125 (88.7)	81 (86.2)	0.98
None	16 (11.3)	13 (13.8)	0.812
1	20 (14.2)	23 (24.5)	
2	20 (14.2)	12 (12.8)	
3+	85 (60.3)	46 (48.9)	
Pentavalent fully vaccinated for age‡			
<1 year old	63 (75.0)	54 (77.1)	0.653
≥1 year old	51 (89.5)	18 (75.0)	0.133
PCV doses received			
*≥*1 dose of PCV	108 (76.6)	74 (78.7)	0.678
None	33 (23.4)	20 (21.3)	0.964
1	20 (14.2)	20 (21.3)	
2	18 (12.8)	13 (13.8)	
3+	70 (49.6)	41 (43.6)	
PCV fully vaccinated for age‡			
<1 year old	62 (73.8)	50 (71.4)	0.877
*≥*1 year old	35 (61.4)	13 (54.2)	0.118
MUAC <11.5 cm (children ≥6 months)	26 (27.7)	10 (20.4)	0.354
WAZ by WHO standards			
≥−2	75 (51.7)	51 (53.1)	0.539
−3 ≤ to *<*−2	24 (16.6)	20 (20.8)	
<−3	46 (31.7)	25 (26.0)	
WHZ by WHO standards			
≥−2	82 (56.6)	55 (57.3)	0.991
−3 ≤ to *<*−2	25 (17.2)	16 (16.7)	
<−3	38 (26.2)	25 (26.0)	
HAZ by WHO standards			
≥−2	98 (67.6)	78 (81.3)	0.025
−3 ≤ to *<*−2	28 (19.3)	7 (7.3)	
<−3	19 (13.1)	11 (11.5)	
Hypoxemia§	71 (49.0)	62 (64.6)	0.039
Tachypnea¶	127 (87.6)	78 (81.3)	0.396
Tachycardia‖	81 (55.9)	64 (66.7)	0.082
Danger signs			
Head nodding	0 (0)	58 (60.4)	N/A
Central cyanosis	0 (0)	17 (17.7)	
Multiple or prolonged seizures**	0 (0)	17 (17.7)	
Lethargy	0 (0)	18 (18.8)	
Unable feed	0 (0)	32 (33.3)	
Vomiting	0 (0)	6 (6.3)	
Lung sounds			
Rales	106 (73.1)	71 (74.0)	0.951
Audible wheeze	9 (6.2)	4 (4.2)	0.492
Wheeze on auscultation	21 (14.5)	14 (14.6)	0.777
Grunting	82 (56.6)	70 (72.9)	0.005
Nasal flaring	119 (82.1)	83 (86.5)	0.435
Elevated temperature (≥38°C)	57 (39.3)	32 (33.3)	0.831
Leukocytosis††	57 (39.3)	29 (30.5)	0.305
CRP ≥40 mg/L	56 (44.1)	33 (41.3)	0.692
Hemoglobin			
0–7.5 g/dL	33 (22.8)	11 (11.6)	0.085
7.6–13.5 g/dL	107 (73.8)	83 (87.4)	
>13.5 g/dL	5 (3.4)	1 (1.1)	
Antibiotic pretreatment before specimen collection‡‡	26 (17.9)	28 (29.2)	0.069
Serum antibiotic activity	18 (12.4)	23 (24.2)	0.024
Parental report of antibiotics	69 (52.3)	48 (57.8)	0.677
Antibiotics administered at study hospital§§	6 (4.1)	1 (1)	0.133
Duration of illness before admission¶¶			
Median in days (IQR)	5 (3–7)	5 (3–7)	
0–2 days	12 (8.3)	11 (11.5)	0.712
3–5 days	69 (47.9)	47 (49)	
>5 days	63 (43.8)	38 (39.6)	
Duration of hospitalization			
Median in days (IQR)	7 (4, 11)	6 (3.5, 9)	
0–2 days	4 (2.8)	6 (6.3)	0.342
3–5 days	48 (33.1)	35 (36.5)	
>5 days	93 (64.1)	55 (57.3)	
30-day follow-up completed among those discharged alive	127 (94.8)	75 (94.9)	0.901
Died‖‖	14 (9.9)	17 (18.5)	0.060
In hospital	9 (6.4)	16 (17.4)	0.008
After discharge but within 30 days of admission	5 (3.5)	1 (1.1)	0.247

*P value for difference in medians calculated using Kruskal-Wallis test.

†Adjusted for age except for median age and age category variables, where no adjustment was needed.

‡For children <1 year, defined as received at least 1 dose and up-to-date for age based on the child’s age at enrolment, doses received and country schedule (allowing 4-week window each for dose). For children >1 year defined as 3+ doses.

§Oxygen saturation <90% at admission on room air odds ratio (OR) child on oxygen with no room air reading available.

¶Tachypnea: ≥60 breaths (<2 months), ≥50 breaths (2–11 months) and ≥40 breaths (12–59 months).

‖Tachycardia: >160 beats per minute (bpm) (<11 months), >150 bpm (12–35 months) and >140 bpm (36–59 months).

**Multiple or prolonged convulsions (>15 minutes).

††Leukocytosis defined as white blood cell count ≥15,000 per µL.

‡‡Presence of antibiotics by serum, antibiotics at the referral hospital, clinician report of antibiotics before specimen collection or antibiotics before specimen collection based on time of specimen collection and time of antibiotic administration.

§§Reported by clinician.

¶¶Duration of illness defined as number of days of cough, fever, difficulty breathing or wheeze, whichever is longest before date of admission.

‖‖Among those with vital status available (n = 141 for severe cases; n = 92 for very severe cases).

IQR indicates interquartile range; MUAC, midupper arm circumference. N/A indicates not applicable.

### Laboratory Results Among CXR+, HIV− Cases and Controls

#### Blood Culture, Pleural Fluid, Induced Sputum and Lung Aspirates

Twelve cases had bacteremia (5.0%), of which 6 were culture positive for *S. pneumoniae* (3 were PCV-13 type, 6B, 19A and 23F), 5 were culture positive for *H. influenzae* (4 Hib) and 1 was culture positive for nontyphoidal *Salmonella spp.* (Table 3). Of note, 2 Hib and all 3 PCV-13 type pneumococcus blood culture positive cases were reported by parents as fully vaccinated, including one 23-month old with Hib grown from blood culture who died after discharge. MTB culture results were positive in 1 of 223 (0.4%) cases with induced sputum or gastric aspirate specimens collected. A pathogen was identified in 4 of 5 pleural fluid specimens collected within 3 days of enrollment; 2 were positive for *Staphylococcus aureus* by culture and PCR, the third was culture positive for both *Escherichia coli* and group F Streptococcus and the fourth was positive for *S. pneumoniae* by PCR alone (Table 3). Of the 9 lung aspirate samples that were collected within 3 days of enrollment, none were culture positive and no sample remained for PCR testing. Eight of the 9 cases received antibiotics at the hospital before the lung aspirate specimen was collected (range: 26–96 hours since antibiotic administration).

**TABLE 3.** Blood, Induced Sputum and Pleural Fluid Culture Results of HIV-Uninfected, CXR+ Cases

	CXR+ Cases (n = 241)
Blood culture	
Any noncontaminant organism	12 (5.0)
*Streptococcus pneumoniae*	6 (2.5)
*S. pneumoniae*, vaccine type (PCV-13)	3 (1.2)*
*S. pneumoniae*, nonvaccine type (PCV-13)	3 (1.3)
*Haemophilus influenzae*	5 (2.1)
*H. influenzae* type b	4 (1.7)
*H. influenzae* nontype b	1 (0.4)
Nontyphoidal *Salmonella*	1 (0.4)
Contaminants†	17 (7.1)
Induced sputum/gastric aspirate	
*Mycobacterium tuberculosis*‡	1 (0.4)
Pleural fluid culture§	
*Staphylococcus aureus*	2 (0.8)
*Escherichia coli*	1 (0.4)
Group F *Streptococcus*	1 (0.4)
Pleural fluid PCR	
*S. aureus*	2 (0.8)
*S. pneumoniae*	1 (0.4)

*NP serotype used as proxy for blood culture serotype for 2 cases (1 CXR+ case) missing serotype data for blood culture isolate. High concordance between NP and blood culture isolates provided the rationale.

†Blood culture contaminants were defined a priori by standard criteria, and these results were excluded from the etiology analysis.

‡Out of 200 cases with induced sputum results available, an additional 23 cases with only gastric aspirate results available. The 1 positive result was from a gastric aspirate sample.

§Only positive results from 5 cases with pleural fluid samples obtained within 3 days of enrolment were used to inform the case’s individual etiology in the PERCH integrated analysis. These results were not extrapolated to other cases. Of the 2 pleural fluid specimens collected outside this window, 1 (collected 4 days after enrolment) was positive for *Moraxella catarrhalis* by culture and for *M. catarrhalis*, *S. pneumoniae* and cytomegalovirus (CMV) by PCR; the other (collected 14 days after enrolment) was positive for *S. aureus* by culture and for *S. aureus*, CMV and respiratory syncytial virus by PCR.

#### NP/OP Swab PCR

**FIGURE 2.**
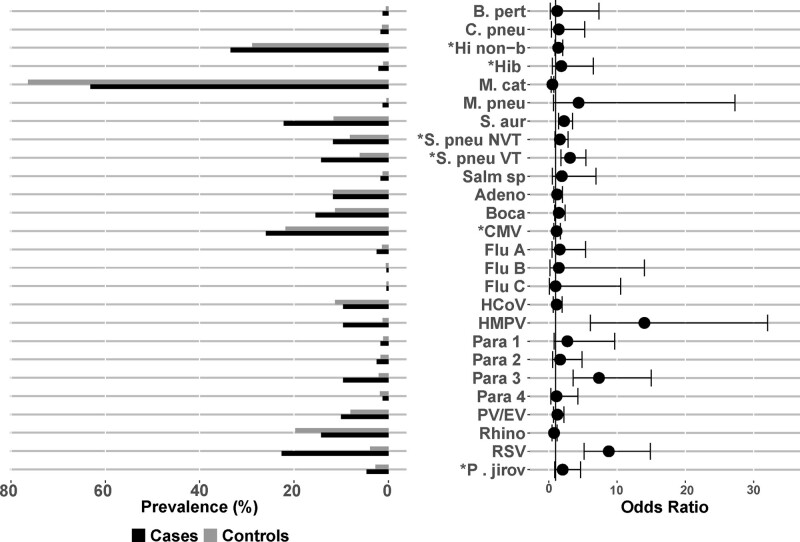
NP/OP PCR prevalence and odds ratios by pathogen among CXR+, HIV-uninfected cases and HIV-uninfected controls. Pathogens are ordered alphabetically among bacteria and then viruses and fungi. *Prevalence defined using NP/OP PCR density thresholds for 4 pathogens: *P. jirovecii*, 4 log_10_ copies/mL; *H. influenzae*, 5.9 log_10_ copies/mL; CMV, 4.9 log_10_ copies/mL; *S. pneumoniae*, 6.9 log_10_ copies/mL). NP/OP PCR results based on positivity are given in Table, Supplementary Digital Content 4, http://links.lww.com/INF/E14. Odds ratios adjusted for age (months), site and presence of other pathogens detected by NP/OP PCR. Adeno indicates Adenovirus; B. pert, *Bordetella pertussis*; Boca, Human bocavirus; C. pneu, *Chlamydophila pneumoniae*; CMV, cytomegalovirus; Flu, influenza virus; HCoV, human coronavirus; Hib, *Haemophilus influenzae* type b; Hi non-b, *Haemophilus influenzae* non-b; HMPV, human metapneumovirus A/B; Legio, Legionella; M. cat, *Moraxella catarrhalis*; M. pneu, *Mycoplasma pneumoniae*; NVT, non PCV13 vaccine type; Para, Parainfluenza virus; P. jirov, *Pneumocystis jirovecii*; PV/EV, parechovirus/enterovirus; Rhino, rhinovirus; RSV, Respiratory syncytial virus A/B; S. aur, *Staphylococcus aureus*; S. pneu, *Streptococcus pneumoniae*; Salm sp, *Salmonella* spp.; VT, PCV-13 vaccine type.

The organisms detected in the NP/OP swab by PCR with a higher odds of detection among cases compared with controls after adjusting for age and codetection of other pathogens were human metapneumovirus (HMPV) (9.6% vs. 1.2%; OR = 13.5), RSV (22.6% vs. 3.9%; OR = 8.6), parainfluenza virus (PIV) type 3 (9.6% vs. 2.1%; OR = 7.0), high density (>6.9 log_10_ copies/mL) PCV-13 type *S. pneumoniae* (25.9% vs. 15.6%; OR = 2.2) and *S. aureus* (22.2% vs. 11.6%; OR = 2.2) (Fig. 2 and Table, Supplemental Digital Content 4, http://links.lww.com/INF/E14). RSV detection was highest among children 1–5 months (36.7% among cases, 4.1% among controls), and PIV type 3 detection was highest among cases 6–11 months (17% among cases) (Table, Supplemental Digital Content 5, http://links.lww.com/INF/E15).

### PIA Etiologic Results for CXR+, HIV− Cases

**FIGURE 3.**
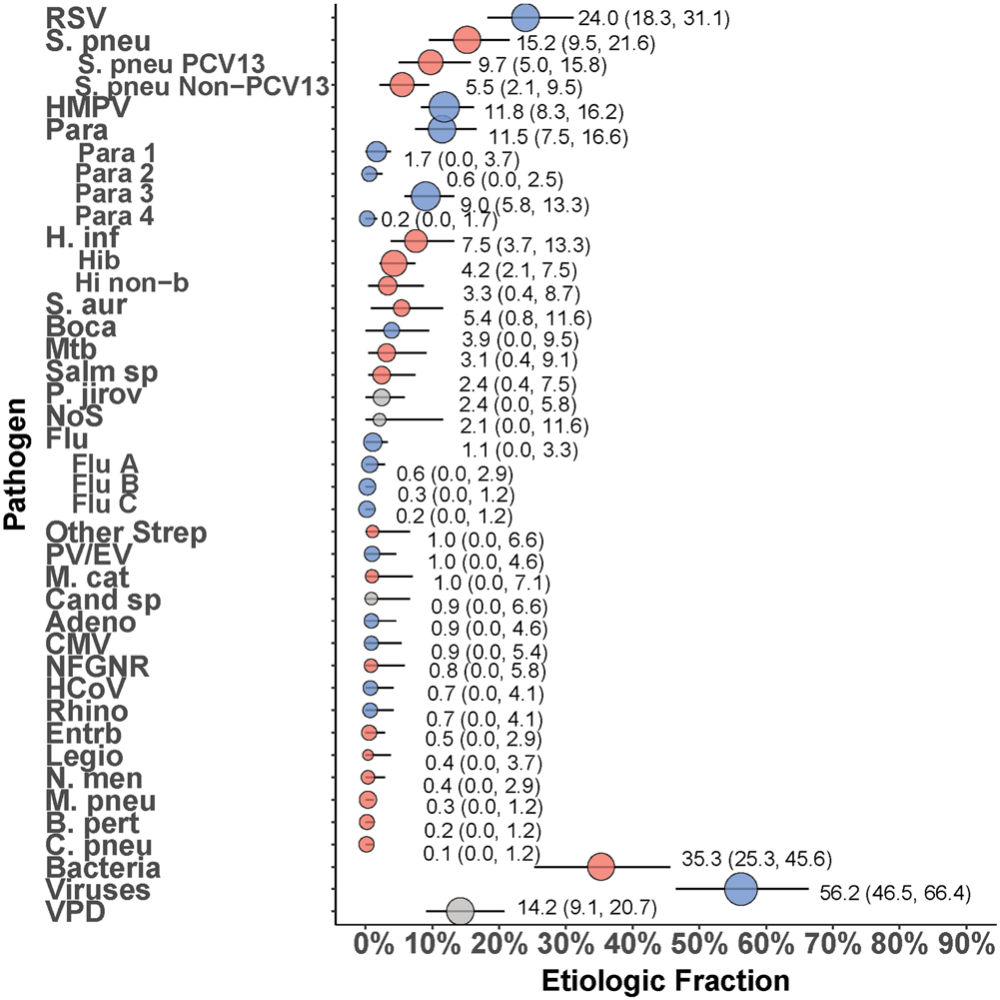
Etiology of severe and very severe pneumonia among CXR+, HIV-uninfected cases using the integrated analysis method. Other Strep includes *Streptococcus pyogenes* and *Enterococcus faecium*. NFGNR includes *Acinetobacter* spp. and *Pseudomonas* spp. *Enterobacteriaceae* includes *E. coli*, *Enterobacter* spp., and *Klebsiella* spp., excluding mixed gram-negative rods. Vaccine-preventable disease includes *S. pneumoniae* PCV-13 type, *H. influenzae* type b and *B. pertussis*. CXR+ defined as consolidation and/or other infiltrate on chest radiograph. Bacterial summary excludes MTB. Pathogens estimated at the subspecies level are presented grouped and disaggregated (Parainfluenza virus type 1, 2, 3 and 4; *S. pneumoniae* PCV 13 and *S. pneumoniae* non-PCV 13 types; *H. influenzae* type b and *H. influenzae* non-b; influenza A, B, and C). Description of symbols: Color of symbols: Red = bacteria; Blue = viruses; Gray = other. Line represents the 95% credible interval. The size of the symbol is scaled based on the ratio of the estimated etiologic fraction to its standard error. Of 2 identical etiologic fraction estimates, the estimate associated with a larger symbol is more informed by the data than the priors. Adeno indicates Adenovirus; B. pert, *Bordetella pertussis*; Boca, Human bocavirus; C. pneu, *Chlamydophila pneumoniae*; Cand sp, Candida species; CMV, cytomegalovirus; Entrb, Enterobacteriaceae; Flu, influenza virus A, B and C; H. inf, *Haemophilus influenzae*; HCoV, Coronavirus; HMPV, human metapneumovirus A/B; Legio, Legionella species; M. cat, *Moraxella catarrhalis*; M. pneu, *Mycoplasma pneumoniae*; Mtb, *Mycobacterium tuberculosis*; NFGNR, nonfermentative gram-negative rods; N. men, *Neisseria meningitidis*; NoS, not otherwise specified (ie, pathogens not tested for); P. jirov, *Pneumocystis jirovecii*; Para, parainfluenza virus types 1, 2, 3 and 4; PV/EV, parechovirus/enterovirus; Rhino, human rhinovirus; RSV, respiratory syncytial virus A/B; S. aur, *Staphylococcus aureus*; S. pneu, *Streptococcus pneumoniae*; Salm sp, *Salmonella* spp.; VPD, vaccine-preventable disease.

In CXR+, HIV− cases, the predominant causal pathogens were RSV (24.0%; 95% CrI: 18.3%–31.1%), *S. pneumoniae* (15.2%; 95% CrI: 9.5–21.6), of which PCV-13 type made up the majority (9.7%; 95% CrI: 5.0–15.8), HMPV (11.8%; 95% CrI: 8.3%–16.2%) and PIV (11.5%; 95% CrI: 7.5–16.6), predominated by PIV type 3 (9.0%; 95% CI: 5.8%–13.3%) (Fig. 3). Together, these pathogens accounted for 62.5% of cases.

Age-related patterns in etiology are shown in Figure, Supplemental Digital Content 6, http://links.lww.com/INF/E16. The dominance of RSV was most marked in the 1- to 11-month age group (32.5% in cases 1–11 months vs. 7.9% in cases ≥12 months). The ratio of PCV-13 type to nonvaccine type (NVT) *S. pneumoniae* also differed by age: among ≥12 months of age, *S. pneumoniae* cases were predominantly PCV-13 type (20.8% vs. 1.8% NVT), while those 1–11 months of age were more evenly distributed NVT (PCV-13 type = 3.8% vs. NVT = 7.4%). Pneumonia attributed to PIV type 3 occurred almost exclusively in cases 1–11 months of age (12.8% vs. 1.9%). Among cases 1–11 months, viral etiologies dominated (65.4% viral vs. 29.6% bacterial); among cases ≥12 months, 45.9% had bacterial and 39.1% had viral etiologies. The viral etiologies in cases 1–11 months comprised primarily by RSV, HMPV and PIV type 3, which together accounted for 58.1% of the cases in this age group.

Cases with moderate-to-severe wasting (WHZ < −2) or stunting (HAZ < −2) were less likely to have pneumonia caused by RSV compared with other children (11.5% vs. 27.3% for those with wasting and 6.1% vs. 28.5% for those with stunting, Figure, Supplemental Digital Content 7, http://links.lww.com/INF/E17 and Figure, Supplemental Digital Content 8, http://links.lww.com/INF/E18). There were similar findings for HMPV (3.7% vs. 14.4% for those with and without moderate-to-severe wasting and 1.1% vs. 11.1% for those with and without moderate-to-severe stunting). Compared with those without moderate-to-severe wasting, the etiology of pneumonia in those with moderate-to-severe wasting was more commonly due to *H. influenzae* (20.9% vs. 2.9%), *S. aureus* (13.5% vs. 2.6%) and MTB (6.9% vs. 0.5%). The etiology of pneumonia in those with moderate-to-severe stunting was more evenly distributed across pathogens, but with a trend toward more bacterial than viral pathogens. In children with moderate-to-severe stunting, the most common causes were *S. pneumoniae* (11.2%), *H. influenzae* (9.7%), MTB (6.9%), *S. aureus* (6.6%), parainfluenza type 3 (6.5%), *Salmonella* spp. (6.4%) and RSV (6.1%).

### Laboratory and Integrated Etiology Results for CXR+, HIV− Cases Who Died

One case who died had a Hib-positive blood culture, and a higher proportion of fatal cases had high-density *P. jirovecii* detected from NP/OP compared with cases who survived [4/31 (12.9%) vs. 6/200 (3.0%), *P* = 0.012; Table, Supplemental Digital Content 9, http://links.lww.com/INF/E19]. All 4 fatal cases who had *P. jirovecii* detected from NP samples above the density threshold had unknown HIV status. Conversely, *H. influenzae* that was not type b was detected less frequently from NP/OP of cases who died compared with those who survived [117/200 (57.9%) vs. 11/31 (35.5%), *P* = 0.016]. The etiologic distribution of pathogens in the 31 cases who died in the hospital or within 30 days of hospital admission was different than in the 202 cases who survived to that point (Figure, Supplemental Digital Content 10, http://links.lww.com/INF/E20). Although the number of fatal cases was small and the CrIs overlapped for individual pathogens, *S. aureus*, *P. jirovecii* and Hib together accounted for 39.5% of fatal cases, and only 6.9% of cases who survived. Conversely, RSV, HMPV and PIV-3 together comprised 48% of cases who survived and only 6.5% of cases who died. Overall, cases who survived had etiologies that were predominantly viral (59.9%; 95% CrI: 49.5–70.0), whereas viral etiologies only comprised 31.9% (95% CrI: 9.7–61.3) of cases who died. The CFR for cases estimated to be caused by RSV was 1.5% (95% CrI: 0.0–6.9), compared with 16.2% (95% CrI: 14.6–17.8) for cases estimated to be caused by a non-RSV pathogen.

### Secondary Analyses of Children Who Had Normal or Missing CXR Findings or Were HIV+

#### Comparison of HIV- Children [not HIV−Children] With a Positive CXR to Those With Normal or Missing CXRs

Compared with cases with normal CXRs, and after adjusting for age, CXR+ cases were less likely to meet criteria for very severe pneumonia (39.8% vs. 57.4%, *P* < 0.001) and were more likely to be severely underweight [weight for age Z score (WAZ) <−3, 29.5% vs. 11.5%, *P* < 0.001], severely wasted (WHZ <−3 26.1% vs. 15.2%, *P* = 0.003) moderate to severely stunted (HAZ <−2, 26.6% vs. 13.3%, *P* < 0.001), hypoxemic (55.2% vs. 35.0%, *P* < 0.001), to have leukocytosis (35.9% vs. 21.6%, *P* < 0.001), elevated CRP (43.0% vs. 25.%, *P* < 0.001) and to have rales on auscultation (73.4% vs. 55.2%, *P* < 0.001) (Table, Supplemental Digital Content 11, http://links.lww.com/INF/E21). There were no differences between CXR+ and CXR normal cases with respect to mortality (CFR = 13.3% vs. 8.9%, *P* = 0.15). Compared with cases who did not have a CXR taken within 72 hours of admission (CXR missing), CXR+ cases were less likely to meet the criteria for very severe pneumonia (39.8% vs. 84.4%, *P* < 0.001) and were less likely to die (13.3% vs. 56.8%, *P* < 0.001).

#### Cases With HIV Infection

Among the 21 (HIV+) cases, 18 (85.7%) had a CXR result, with 12 (57.1%) CXR+, and 3 (14.3%) each CXR normal or CXR uninterpretable. Among the 12 CXR+, 7 (58.3%) had very severe pneumonia. There were 8 blood culture positive results among the 21 HIV+ cases: 1 *Pseudomonas aeruginosa*, 1 *S. aureus* and 6 *S. pneumoniae*, 5 of which were PCV-13 vaccine type. The CFR for HIV+ cases was 35.0% (7 of 20 with follow-up data) within 30 days of admission. Moreover, 6.1% of all fatal cases (7/115) were HIV+ compared with 2.4% (13/538) of surviving cases. CXR+ HIV-infected and HIV-exposed cases had a trend toward higher rates of underweight, wasting and stunting compared with HIV-uninfected/unexposed cases that was not statistically significant (Table, Supplemental Digital Content 3, http://links.lww.com/INF/E13 and Table 1). Mortality was higher in malnourished cases regardless of HIV status, but cases who were both HIV infected and malnourished had the highest case fatality (Table, Supplemental Digital Content 12, http://links.lww.com/INF/E22).

## DISCUSSION

Using a nested partially latent class analysis of pneumonia etiology that integrated results from multiple case and control specimens, the PERCH study in Bamako, Mali, demonstrated that RSV was the principal cause (24% overall) of severe and very severe pneumonia among hospitalized HIV− children 1–59 months of age with a positive CXR. Among children 1–11 months of age, who comprised 65% of cases, most (65%) pneumonia episodes were attributed to viruses, and 33% were attributable to RSV. Despite the low CFR (1.5%) for RSV, its high etiologic fraction translates into a substantial burden of morbidity and mortality and suggests that interventions to prevent and treat these infections could have a considerable impact. Vaccines appear to be the most promising strategy for RSV disease control in low resource settings with several candidates in the clinical development pipeline.^21^ Two-thirds of CXR+/HIV− cases with RSV detected by PCR in NP/OP specimens were less than 6 months of age, suggesting that maternal immunization could be a useful prevention strategy. However, in the Malian setting, persistence of maternal antibody is likely to be short-lived^22,23^ and wanes after the first 4 months of life.^23^ Because RSV remains 1 of the top 3 causes of severe pneumonia in children beyond 4 months of age, infant vaccination might be a useful adjunct for continued protection against RSV disease that occurs outside of early infancy.

Despite moderately high immunization coverage in our study, vaccine-preventable causes (PCV-13 type *S. pneumoniae* and Hib) together accounted for 14% of HIV− CXR+ pneumonia cases. Vaccination status was ascertained by a combination of vaccination cards and maternal report with only 26% of cases and 64% of controls having vaccine cards available, so it is possible that misclassification occurred; however, our coverage rates are consistent with national estimates.^24^ PCV-13 was introduced into Mali’s Expanded Programme on Immunization program the year before PERCH enrollment, and younger children therefore had a greater opportunity to be fully vaccinated with PCV. This is reflected by the higher ratio of NVT:PCV-13 type *S. pneumoniae* cases identified in children <1 year of age compared with those ≥1 year. Nonetheless, our findings that ≈25% of infants were not fully vaccinated against *S. pneumoniae* and Hib and that both pathogens continue to cause morbidity and mortality in Malian infants with severe pneumonia indicate that efforts to improve vaccine coverage must be a high priority.

PERCH uncovered an exceptionally high CFR within 30 days of admission (13.3%) in Mali, with *S. aureus*, *P. jirovecii* and Hib comprising 40% of fatal cases. This finding is consistent with Mali’s rank as the country with the highest under-5 mortality rate at the start of PERCH in 2011^25^ and warrants careful examination, particularly in the face of low HIV prevalence and moderately high reported uptake of vaccines against bacteria known to cause pneumonia. Malnutrition likely contributes to mortality as it was more common among cases who died. Other contributing factors are system-based weaknesses, such as overcrowding that sometimes requires multiple infants to share a bed and a single oxygen source, insecure oxygen supply and lack of ventilatory support. The CFRs reported here likely underestimate the true pneumonia mortality burden in this setting as well as the contribution of pathogens associated with fatal pneumonias because (1) the standard of care was enhanced by the addition of study-specific resources such as provision of pulse oximetry, supplementary oxygen and oxygen delivery supplies, antibiotics and intravenous fluids free of charge; (2) cases with a missing CXR, not included in the primary analysis, had higher CFR; and (3) some cases died too quickly or were too ill to be enrolled in the study (n = 5). The ability of PERCH to illuminate these issues for public health partners is a critical step toward positive action.

A limitation of the study is that HIV testing was refused in roughly one-third of cases, and some cases with unknown HIV status were therefore likely incorrectly classified as HIV negative in the primary analyses. If the proportion of cases with HIV-unknown status who were misclassified as negative was the same as the proportion of cases who tested HIV positive (4.5%), then 3–4 HIV-infected children would have been misclassified as HIV uninfected in the primary analysis. *P. jirovecii*, an organism more frequently detected in HIV-infected individuals,^26^ was detected at high densities in the NP specimens of 4 CXR+ cases who died, all of whom had unknown HIV status, raising the possibility that these cases may have been HIV infected. If refusal of consent was more likely among infected cases, then the number of those misclassified would be higher. The higher prevalence of moderate-to-severe wasting among cases with unknown HIV status (52%) compared with HIV negative (39%) also suggests that some HIV-infected cases may have been misclassified.

Coinciding with the PERCH study in Mali was another study of the etiology of pneumonia in children hospitalized at HGT; it was a multicenter case-control study, part of the Global Approach to Biological Research, Infectious diseases and Epidemics in Low-income countries (GABRIEL) network.^27^ GABRIEL enrolled pneumonia cases during an 18-month period that overlapped with PERCH for 1 year (2012). Like PERCH, GABRIEL found that *S. pneumoniae*, RSV and HMPV were the most important etiologies associated with pneumonia. However, *S. pneumoniae* was the leading pathogen in GABRIEL, while RSV was the leading pathogen in PERCH. Discrepancies in the importance of several other agents were also apparent. Methodologic differences between the 2 studies may explain the differences in findings. GABRIEL cases were older [median age: 12 months (GABRIEL) vs. 8 months (PERCH)] and included those with pneumonia of any severity. GABRIEL excluded participants who presented with wheezing, whereas PERCH only excluded wheezing children who were responsive to a bronchodilator challenge. The burden of RSV in PERCH was observed to be higher in 2013 than in 2012, which may have contributed to increased detection of RSV in PERCH. Another difference is that GABRIEL recruited outpatients or children who were hospitalized for surgery as controls. These children may not be representative of the general population from which cases were drawn and may have had nosocomial exposure to respiratory pathogens such as RSV or antibiotic treatment, possibly decreasing *S. pneumoniae* colonization. This may explain why GABRIEL found higher RSV infection (6.1% vs. 3.9%) and lower pneumococcal colonization (48.0% vs. 79.1%) in controls than PERCH, in turn decreasing the OR for RSV and increasing the OR for *S. pneumoniae* pneumonia. Finally, GABRIEL used logistic regression techniques to estimate attributable fractions, whereas PERCH used the Bayesian models described herein. These methodologic variations could result in different pathogen prevalence, especially for those that vary by season, age, disease severity and/or disease manifestations among cases.

The PERCH study must be understood in the context of its strengths and weaknesses. In addition to a high proportion of participants with unknown HIV status, other limitations included challenges in collecting specimens before the administration of antibiotics, the inability to obtain a sample from the site of infection (the lung) from almost all cases, the exclusion of cases from the primary analysis who died before a CXR could be obtained and the exclusion of children who died before they could be enrolled in the study.

## CONCLUSIONS

The PERCH study provides unique information about the etiology and clinical outcomes of severe and very severe pneumonia among Malian infants and young children. Our findings, particularly the predominance of RSV, high rates of malnutrition in children hospitalized with pneumonia and the high overall CFR, can inform public health stakeholders in Mali and other low resource African countries with low HIV prevalence. These data highlight a role for new, effective interventions, such as RSV vaccines, but they also underscore the serious need for sustained and improved coverage of existing vaccines against Hib and *S. pneumoniae* and for the strengthening of health care delivery to ensure prompt administration of life-saving nutritional interventions and pneumonia treatment.

## ACKNOWLEDGMENTS

The authors thank the participants and their families for their time and cooperation. We also acknowledge the support of the l’Hôpital Gabriel Touré (HGT) administration and the Ministry of Health of Mali. We recognize the efforts of the following groups during the study development, conduct and analysis phases: Pneumonia Methods Working Group, Pneumonia Etiology Research for Child Health (PERCH) Expert Group, PERCH contributors and the PERCH Chest Radiograph Reading Panel. We also acknowledge the substantial contributions of all members of the PERCH Study Group. Members of the PERCH Study Group: Johns Hopkins Bloomberg School of Public Health, Baltimore, Maryland: Orin S. Levine (Former PI, current affiliation Bill & Melinda Gates Foundation, Seattle, Washington), Andrea N. DeLuca, Amanda J. Driscoll, Nicholas Fancourt, Wei Fu, E. Wangeci Kagucia, Ruth A. Karron, Mengying Li, Daniel E. Park, Qiyuan Shi, Zhenke Wu, Scott L. Zeger; The Emmes Corporation, Rockville, Maryland: Nora L. Watson; Nuffield Department of Clinical Medicine, University of Oxford, United Kingdom: Jane Crawley; Medical Research Council, Basse, The Gambia: Stephen R. C. Howie (site PI); KEMRI-Wellcome Trust Research Programme, Kilifi, Kenya: J. Anthony G. Scott (site PI and PERCH co-PI, joint affiliation with London School of Hygiene and Tropical Medicine, London, UK); Medical Research Council: Respiratory and Meningeal Pathogens Research Unit and Department of Science and Technology/National Research Foundation: Vaccine Preventable Diseases, University of the Witwatersrand, Johannesburg, South Africa: Shabir A. Madhi (site PI); International Centre for Diarrhoeal Disease Research, Bangladesh (icddr,b): W. Abdullah Brooks (site PI); Boston University School of Public Health, Boston, Massachusetts and University Teaching Hospital, Lusaka, Zambia: Donald M. Thea (site PI); Thailand Ministry of Public Health—U.S. CDC Collaboration, Nonthaburi, Thailand: Henry C. Baggett (site PI), Susan A. Maloney (former site PI); Canterbury Health Laboratories, Christchurch, New Zealand: Trevor P. Anderson, Joanne Mitchell, Shalika Jayawardena, Rose Watt.

## Supplementary Material


