# Supplementary material for: The Etiology of Childhood Pneumonia in Mali: Findings From the Pneumonia Etiology Research for Child Health (PERCH) Study
Source: Pediatr Infect Dis J. 2021 Aug 25;40(9):S18–28. doi: 10.1097/INF.0000000000002767 (PMC8448406; doi:10.1097/INF.0000000000002767)
Supplement: Supplementary file 1 [file inf-40-s18-s001.docx]

**Supplemental Digital Content 1.**

**METHODS**

**Case and Control Identification**

Cases

Cases were selected from among children being hospitalized at l’Hôpital Gabriel Touré (HGT). HGT houses 66 general pediatric inpatient beds and 35 pediatric intensive care beds. The estimated 17,500 annual visits to the emergency department (ED) and outpatient department (OPD) among children younger than 5 years of age result in approximately 7,070 hospitalizations each year. During the pneumonia season, due to increased demand, multiple patients can occupy a single hospital bed. We enrolled a maximum of 4 (year 1) or 6 (year 2) consecutive patients every other day using a schedule of one or two 8-hour shifts (8 am to 4 pm and/or 4 pm to midnight) that repeated in 4-week cycles.

Controls

To select controls, the study team arrived at the designated quartier and randomly selected a direction and distance to travel from the chief’s home (presumed to be at the center of the neighborhood). After traveling the selected distance, the study team visited the first house on the left to find a control in the assigned age group, moving leftward from house-to-house until an age-eligible child was identified. If the caregiver consented, the child was enrolled and had data and specimens collected at the home. Once an eligible child was enrolled, this process was repeated if another child was to be enrolled from the same quartier.

**HIV testing and classification** Parent/guardians of cases and controls were asked about the participant’s mother’s HIV status during her pregnancy. The information was recorded based on self-report or medical record, if available. Participants underwent HIV testing if their parent/guardian signed a separate consent document for HIV testing according to the policies of the Malian Ethics Committee. Participants were first tested with an HIV antibody test. Those fewer than 12 months of age with a positive antibody result were re-tested with a confirmatory PCR test. Cases and controls were considered HIV-infected (HIV+) if they had a positive PCR result or if they were 12 months of age or older and had a positive antibody test result. Cases and controls with missing antibody results or those less than 12 months who were antibody positive but missing PCR results, were classified as having unknown HIV status, regardless of the child’s or mother’s reported status. Cases and controls were considered HIV-uninfected (HIV-) in the primary analysis if their antibody or PCR test results were negative (HIV test-negative) or if their HIV status was unknown. Participants were considered HIV-exposed if they were HIV antibody test positive but PCR negative or were born to a mother who reported being HIV-infected during her pregnancy, unless the child was <7 months of age and had a negative antibody test. HIV exposure was classified as unknown if results were missing for HIV antibody or PCR (for antibody positive children <12 months) tests, or the mother’s HIV status during pregnancy was unknown.

**PERCH Integrated Analysis (PIA) Methods**

The PIA accounts for imperfect sensitivity of each test/pathogen measurement by using *a priori* estimates of their sensitivity (i.e., estimates regarding the plausibility range of sensitivity which varied by laboratory test method and pathogen, **Table,** and Appendix Section III B of (3). As a Bayesian analysis, the potential pathogens and their starting ‘prior’ etiologic fraction values were specified *a priori,* which favored no pathogen over another (i.e., were ‘uniform’). The pathogens selected for inclusion in the analysis included any non-contaminant bacteria detected by culture in blood at any of the PERCH sites, regardless of whether it was observed at the Mali site specifically, MTB, and all of the multiplex quantitative PCR pathogens except those considered invalid because of poor assay specificity (*Klebsiella* *pneumoniae* (21) and *M. catarrhalis*). A category called ‘Pathogens Not Otherwise Specified’ (NoS) was included to estimate the fraction of pneumonia caused by pathogens not tested for or not observed. A child negative for all pathogens would still be assigned an etiology, which would be either one of the explicitly estimated pathogens (implying a ‘false negative’, accounting for imperfect sensitivity of certain measurements) or NoS.

Separate etiology models were run with additional covariates, including vital status within 30 days of admission and nutritional status (one model included wasting, defined as a weight for height z-score <-2, the other included stunting, defined as a height for age z-score <-2). These models link the covariate value of the child (e.g. died or alive) with the etiology assignment at each iteration of the analysis.

All analyses were adjusted for age (<1 vs ≥1 year) to account for differences in pathogen prevalence by age. For results stratified by vital status, the test results from all controls were used. However, for analyses stratified by age or nutritional status, only data from controls representative of that age group were used.

**Supplemental Digital Content 1, Table. Integrated etiology analysis input values for sensitivity and specificity of laboratory test measures**

For more information about the sensitivity priors in the PERCH Integrated Etiology Analysis refer to [Appendix](https://www.thelancet.com/cms/10.1016/S0140-6736(19)30721-4/attachment/6303e6bf-f391-4045-b893-0a85966ff700/mmc1.pdf) Section III B 6 in [The PERCH Study Group. Causes of severe pneumonia requiring hospital admission in children without HIV infection from Africa and Asia: the PERCH multi-country case-control study. Lancet. 2019; 6736(19):1-23](https://www.thelancet.com/journals/lancet/article/PIIS0140-6736(19)30721-4/fulltext#section-7c530872-6235-4433-899c-b3f276970189).

|  |  | **Sensitivity Prior^a^** | |  |
| --- | --- | --- | --- | --- |
| **Specimen/test** | **Pathogen** | **Base** | **Reduced** | **Specificity** |
| Blood cultures^c^ | *-Streptococcus pneumoniae*  *-Haemophilus influenzae* | 5-20% | 1-13% | 100% |
|  | *-Moraxella catarrhalis*  *-Staphylococcus aureus*  -Nonfermentative gram-negative rods  -Candida species  -Non-pneumococcal streptococci, including enterococci | 5-15% | 1-10% |  |
|  | Salmonella species  Enterobacteriaceae  *Neisseria meningitidis* | 10-50% | 1-34% |  |
| NP/OP PCR | *-Streptococcus pneumoniae*  *-Haemophilus influenzae* | 50-90% | 15-55% | 1 − Control prevalence (ref SDC Table 4) |
|  | *-*Salmonella species  -Legionella species | 0.5-90% | 0.5-90% |  |
|  | -All other PCR targets | 50-90% | 50-90% |  |
| Whole blood PCR | *- Streptococcus pneumoniae* | 12-65% | 12-65% | 1 − Control prevalence (ref SDC Table 4) |
| Induced sputum | -*Mycobacterium tuberculosis* | 10-30% | 10-30% | 100% |

Abbreviation: SDC, Supplemental Digital Content.

a. Background information supporting choice of sensitivity priors provided in the all-site PERCH paper (see above).

b. Base: > 1.5 mL blood culture volume (blood culture only) and no evidence of prior antibiotic exposure. Reduced: < 1.5 mL or evidence of prior antibiotic exposure.

c. Direct evidence of the diagnostic sensitivity for *Streptococcus pneumoniae* and *Haemophilus influenzae* from vaccine probe studies. For all other pathogens we set the base blood culture sensitivity prior to 5-15%, with the exception of Salmonella species, Enterobacteriaceae and *Neisseria meningitidis*, for which we selected wider priors (10-50%) to reflect their greater uncertainty.
