# Supplementary material for: The Etiology of Childhood Pneumonia in Mali: Findings From the Pneumonia Etiology Research for Child Health (PERCH) Study
Source: Pediatr Infect Dis J. 2021 Aug 25;40(9):S18–28. doi: 10.1097/INF.0000000000002767 (PMC8448406; doi:10.1097/INF.0000000000002767)
Supplement: Supplementary file 2 [file inf-40-s18-s002.docx]

Supplemental Digital Content 2, Table: Specimen Collection by Case-Control Status

|  | **All Cases**  **(N=653)** | **CXR+ Cases**  **(N=241)** | **Controls**  **(N=725)** |
| --- | --- | --- | --- |
| **All** | N=653 | N=241 | N=725 |
|  | n (%) | n (%) | n (%) |
| **NP/OP VTM swab** | 653 (100) | 241 (100) | 725 (100) |
| **NP STGG swab** | 616 (94.3) | 229 (95) | 687 (94.8) |
| **Blood Culture** | 653 (100) | 241 (100) | N/A |
| **Plain/red top tube** | 651 (99.7) | 240 (99.6) | 698 (96.3) |
| **EDTA tube 1 (CBC)** | 649 (99.4) | 241 (100) | 720 (99.3) |
| **Serum** | 653 (100) | 241 (100) | N/A |
| **Convalescent serum** | 465 (71.2) | 180 (74.7) | N/A |
| **Induced Sputum** | 529 (81.0) | 202 (83.8) | N/A |
| **Severe cases^a^** | 287 (91.4) | 129 (89) | N/A |
| **Very severe cases^b^** | 242 (71.4) | 73 (76.0) | N/A |
| **Urine** | 485 (74.3) | 188 (78.0) | 408 (56.3) |
| **Convalescent urine** | 315 (48.2) | 130 (53.9) | N/A |
| **Lung Aspirate** | 12 (1.8) | 10 (4.1) | N/A |
| **Severe cases^a^** | 7 (2.2) | 7 (4.8) | N/A |
| **Very severe cases^b^** | 5 (1.5) | 3 (3.1) | N/A |
| **Pleural fluid** | 8 (1.2) | 7 (2.9) | N/A |
| **Severe cases^a^** | 6 (1.9) | 6 (4.1) | N/A |
| **Very severe cases^b^** | 2 (0.6) | 1 (1.0) | N/A |
| ^a^ percentage calculated among severe cases | | | |
| ^b^ percentage calculated among very severe cases | | | |
