# Supplementary material for: The Etiology of Childhood Pneumonia in Mali: Findings From the Pneumonia Etiology Research for Child Health (PERCH) Study
Source: Pediatr Infect Dis J. 2021 Aug 25;40(9):S18–28. doi: 10.1097/INF.0000000000002767 (PMC8448406; doi:10.1097/INF.0000000000002767)
Supplement: Supplementary file 3 [file inf-40-s18-s003.docx]

**Supplemental Digital Content 3.**

**Table. Proportion of malnourished cases by HIV infection and exposure status (n=253 CXR+ cases)**

|  | **HIV infection status** | | | | | **HIV exposure status** | | | | |
| --- | --- | --- | --- | --- | --- | --- | --- | --- | --- | --- |
|  | **Infected^1^** | **Test-negative** | **Un-known** | **HIV-positive vs. HIV-test negative** | **HIV-unknown vs. HIV-test negative** | **Test negative and exposed** | **Test negative and un-exposed** | **Un-known** | **Test negative/ exposed vs. un-exposed** | **Test negative exposed vs. unknown exposure** |
|  | **(N=12)** | **(N=164)** | **(N=77)** |  |  | **(N=6)** | **(N=158)** | **(N=77)** |  |  |
|  | **n (%)** | **n (%)** | **n (%)** | **p-value^1^** | **p-value** | **n (%)** | **n (%)** | **n (%)** | **p-value^1^** | **p-value** |
| Weight for age <-2 z scores | 9 (75.0) | 76 (46.3) | 39 (50.7) | 0.053 | 0.476 | 3 (50.0) | 73 (46.2) | 39 (50.7) | 0.855 | 0.522 |
| Weight for height <-2 z scores | 8 (66.7) | 64 (39.0) | 40 (52.0) | 0.060 | 0.059 | 3 (50.0) | 61 (38.6) | 40 (42.0) | 0.574 | 0.053 |
| Height for age <-2 z scores | 6 (50.0) | 52 (31.7) | 13 (16.9) | 0.193 | **0.016** | 3 (50.0) | 49 (31.0) | 13 (16.9) | 0.327 | **0.021** |
|  | **N=11** | **N=136** | **N=64** |  |  | **N=6** | **N=130** | **N=64** |  |  |
|  | **n (%)** | **n (%)** | **n (%)** | **p-value** | **p-value** | **n (%)** | **n (%)** | **n (%)** | **p-value** | **p-value** |
| MUAC^2^ <11.5cm (children ≥6 months) | 5 (45.5) | 48 (35.3) | 18 (17.3) | 0.500 | 0.145 | 3 (50.0) | 45 (34.6) | 16 (25.0) | 0.441 | 0.175 |

^1^HIV-infected cases were excluded from the primary analysis; HIV test-negative cases are distinguished here from those with unknown HIV status

^2^chi-square p-value

^3^Mid-upper arm circumference
