# Supplementary material for: The Etiology of Childhood Pneumonia in Mali: Findings From the Pneumonia Etiology Research for Child Health (PERCH) Study
Source: Pediatr Infect Dis J. 2021 Aug 25;40(9):S18–28. doi: 10.1097/INF.0000000000002767 (PMC8448406; doi:10.1097/INF.0000000000002767)
Supplement: Supplementary file 4 [file inf-40-s18-s004.docx]

Supplemental Digital Content 4, Table. Detection of Organisms in Nasopharyngeal/Oropharyngeal Specimens Collected from both Cases and Controls^a^

|  | **All Cases** | **CXR+ Cases^b^** | | **Controls** | **Odds Ratios (95% Cis) / P-values^c^** | |
| --- | --- | --- | --- | --- | --- | --- |
|  | **(N=650)** | **All**  **(N=239)** | **Alveolar Consolidation**  **(N=134)** | **(N=724)** | **All Cases vs. All Controls** | **CXR^+^ Cases vs. All Controls** |
| **Pathogen** | | | | | | |
| **Any Pathogen** | 646 (99.4) | 236 (98.7) | 134 (100.0) | 705 (97.4) | 2.80 (0.91, 8.60) | 1.28 (0.35, 4.66) |
| **Any pathogen, with thresholds applied^d^** | 633 (97.4) | 229 (95.8) | 129 (96.3) | 666 (92.0) | 2.10 (1.11, 3.98) | 1.09 (0.48, 2.48) |
| **Bacteria** | | | | | | |
| **Any bacteria** | 589 (90.6) | 213 (89.1) | 123 (91.8) | 683 (94.3) | 0.62 (0.37, 1.04) | 0.40 (0.20, 0.79) |
| **Any bacteria, with thresholds applied for *S. pneumoniae and H. influenzae*** | 532 (81.8) | 192 (80.3) | 111 (82.8) | 609 (84.1) | 1.10 (0.65, 1.85) | 0.68 (0.34, 1.37) |
| ***S. pneumoniae*** | 484 (74.5) | 171 (71.5) | 100 (74.6) | 573 (79.1) | 0.97 (0.71, 1.32) | 0.87 (0.57, 1.33) |
| **>6.9 log10 copies/ml** | 166 (25.5) | 62 (25.9) | 36 (26.9) | 113 (15.6) | **1.87 (1.36, 2.58)** | **2.15 (1.39, 3.32)** |
| **Among those with >6.9 log_10_ copies/ml** |  |  |  |  |  |  |
| **PCV13-type** | 83 (50.0) | 34 (54.8) | 18 (50.0) | 44 (38.9) | **2.31 (1.49, 3.60)** | **3.14 (1.78, 5.54)** |
| **Non PCV13-type** | 69 (41.6) | 28 (45.2) | 18 (50.0) | 59 (52.2) | 1.34 (0.88, 2.05) | 1.59 (0.90, 2.83) |
| ***H. influenzae*** |  |  |  |  |  |  |
| **>=5.9 log10 copies/ml** |  |  |  |  |  |  |
| ***H. influenzae* not type b** | 342 (52.6) | 131 (54.8) | 72 (53.7) | 404 (55.8) | 0.87 (0.67, 1.12) | 0.94 (0.64, 1.36) |
| ***H. influenza*e not type b >=5.9 log10 copies/ml** | 210 (32.3) | 80 (33.5) | 47 (35.1) | 209 (28.9) | 1.27 (0.96, 1.69) | 1.37 (0.91, 2.05) |
| ***H. influenzae* type b** | 15 (2.3) | 7 (2.9) | 4 (3.0) | 14 (1.9) | 1.32 (0.58, 2.99) | 1.63 (0.54, 4.86) |
| ***H. influenzae* type b >=5.9 log10 copies/ml** | 12 (1.8) | 5 (2.1) | 2 (1.5) | 8 (1.1) | 2.03 (0.76, 5.41) | 1.78 (0.49, 6.53) |
| ***S. aureus*** | 133 (20.5) | 53 (22.2) | 34 (25.4) | 84 (11.6) | **1.89 (1.35, 2.64)** | **2.22 (1.42, 3.47)** |
| ***C. pneumoniae*** | 5 (0.8) | 4 (1.7) | 3 (2.2) | 10 (1.4) | 0.67 (0.21, 2.09) | 1.54 (0.41, 5.76) |
| ***M. catarrhalis*** | 419 (64.5) | 151 (63.2) | 88 (65.7) | 553 (76.4) | 0.56 (0.42, 0.73) | 0.50 (0.34, 0.74) |
| ***M. pneumoniae*** | 7 (1.1) | 3 (1.3) | 0 (0) | 3 (0.4) | 3.27 (0.78, 13.71) | 4.29 (0.69, 26.89) |
| **Salmonella species** | 8 (1.2) | 4 (1.7) | 1 (0.7) | 9 (1.2) | 0.88 (0.31, 2.52) | 1.84 (0.50, 6.74) |
| **Legionella** | 0 (0) | 0 (0) | 0 (0) | 0 (0) | ---- | ---- |
| ***B. pertussis*** | 9 (1.4) | 3 (1.3) | 1 (0.7) | 4 (0.6) | 2.23 (0.61, 8.21) | 1.16 (0.19, 7.17) |
| **Virus** | | | | | | |
| **Any virus** | 595 (91.1) | 216 (89.6) | 121 (90.3) | 566 (78.1) | 1.57 (1.05, 2.34) | 1.44 (0.81, 2.56) |
| **Any virus, with thresholds applied for CMV** | 552 (84.9) | 200 (83.7) | 110 (82.1) | 458 (63.3) | 1.77 (1.17, 2.68) | 1.77 (0.98, 3.19) |
| **Adenovirus** | 89 (13.7) | 28 (11.7) | 17 (12.7) | 85 (11.7) | **1.57 (1.10, 2.23)** | 1.22 (0.72, 2.05) |
| **CMV** | 380 (58.5) | 143 (59.8) | 82 (61.2) | 413 (57.0) | 1.25 (0.98, 1.61) | 1.22 (0.85, 1.75) |
| **>4.9 log10 copies/ml** | 179 (27.5) | 62 (25.9) | 34 (25.4) | 158 (21.8) | 1.20 (0.90, 1.60) | 1.10 (0.73, 1.64) |
| **Coronavirus 43** | 23 (3.5) | 3 (1.3) | 1 (0.7) | 30 (4.1) | 1.19 (0.66, 2.15) | 0.44 (0.13, 1.53) |
| **Coronavirus 63** | 20 (3.1) | 7 (2.9) | 3 (2.2) | 32 (4.4) | 1.04 (0.57, 1.93) | 1.29 (0.53, 3.16) |
| **Coronavirus HKU** | 20 (3.1) | 10 (4.2) | 4 (3.0) | 16 (2.2) | 1.55 (0.74, 3.23) | 1.47 (0.56, 3.82) |
| **Coronavirus 229** | 10 (1.5) | 3 (1.3) | 1 (0.7) | 7 (1) | 2.24 (0.74, 6.75) | 2.01 (0.43, 9.44) |
| **HBOV** | 91 (14) | 37 (15.5) | 21 (15.7) | 82 (11.3) | 1.36 (0.94, 1.96) | 1.41 (0.85, 2.33) |
| **HMPV A/B** | 41 (6.3) | 23 (9.6) | 11 (8.2) | 9 (1.2) | **8.36 (3.94, 17.75)** | **13.54 (5.87, 31.20)** |
| **Influenza A** | 15 (2.3) | 6 (2.5) | 3 (2.2) | 10 (1.4) | 1.73 (0.70, 4.29) | 1.56 (0.46, 5.33) |
| **Influenza B** | 7 (1.1) | 1 (0.4) | 1 (0.7) | 4 (0.6) | 3.15 (0.87, 11.48) | 1.44 (0.15, 13.92) |
| **Influenza C** | 3 (0.5) | 1 (0.4) | 1 (0.7) | 3 (0.4) | 0.77 (0.13, 4.42) | 0.91 (0.08, 10.15) |
| **Parainfluenza 1** | 15 (2.3) | 4 (1.7) | 3 (2.2) | 8 (1.1) | **3.58 (1.46, 8.77)** | 2.57 (0.71, 9.33) |
| **Parainfluenza 2** | 13 (2) | 6 (2.5) | 3 (2.2) | 12 (1.7) | 1.11 (0.46, 2.68) | 1.77 (0.59, 5.36) |
| **Parainfluenza 3** | 49 (7.5) | 23 (9.6) | 15 (11.2) | 15 (2.1) | **4.95 (2.66, 9.21)** | **7.04 (3.41, 14.53)** |
| **Parainfluenza 4** | 8 (1.2) | 3 (1.3) | 2 (1.5) | 13 (1.8) | 1.13 (0.44, 2.88) | 1.03 (0.26, 4.01) |
| **PV/EV** | 61 (9.4) | 24 (10) | 15 (11.2) | 58 (8) | 1.33 (0.87, 2.03) | 1.22 (0.68, 2.18) |
| **Rhinovirus** | 113 (17.4) | 34 (14.2) | 17 (12.7) | 143 (19.8) | 0.98 (0.72, 1.35) | 0.74 (0.46, 1.20) |
| **RSV** | 164 (25.2) | 54 (22.6) | 22 (16.4) | 28 (3.9) | **9.45 (6.10, 14.63)** | **8.62 (5.06, 14.67)** |
| **Fungi** | | | | | | |
| ***P. jirovecii*** | 69 (10.6) | 24 (10) | 16 (11.9) | 73 (10.1) | 0.82 (0.54, 1.24) | 0.83 (0.46, 1.50) |
| **>4 log10 copies/ml** | 24 (3.7) | 11 (4.6) | 8 (6.0) | 20 (2.8) | 1.07 (0.54, 2.11) | 1.94 (0.83, 4.54) |
| **Number of organisms, any positivity** | | | | | | |
| Mean (SD) number of organisms | 4.02 (1.51) | 4.02 (1.59) | 4.04 (1.52) | 3.72 (1.56) |  |  |
| Median (IQR) | 4.0 (3.0, 5.0) | 4.0 (3.0, 5.0) | 4.0 (3.0, 5.0) | 4.0 (3.0, 5.0) |  |  |
| **0** | 4 (0.6) | 3 (1.3) | 0 (0) | 19 (2.6) | 0.0045 | 0.1352 |
| **1** | 36 (5.5) | 16 (6.7) | 11 (8.2) | 45 (6.2) |  |  |
| **2** | 56 (8.6) | 17 (7.1) | 8 (6.0) | 92 (12.7) |  |  |
| **3** | 137 (21.1) | 51 (21.3) | 28 (20.9) | 149 (20.6) |  |  |
| **4+** | 417 (64.2) | 152 (63.6) | 87 (64.9) | 419 (57.9) |  |  |
| **Number of organisms, above threshold^b^** | | | | | | |
| Mean (SD) number of organisms | 2.94 (1.40) | 2.95 (1.44) | 2.94 (1.41) | 2.38 (1.42) |  |  |
| Median (IQR) | 3.0 (2.0, 4.0) | 3.0 (2.0, 4.0) | 3.0 (2.0, 4.0) | 2.0 (1.0, 3.0) |  |  |
| **0** | 17 (2.6) | 10 (4.2) | 5 (3.7) | 58 (8.0) | <.0001 | <.0001 |
| **1** | 83 (12.8) | 24 (10.0) | 15 (11.2) | 158 (21.8) |  |  |
| **2** | 160 (24.6) | 62 (25.9) | 32 (23.9) | 185 (25.6) |  |  |
| **3** | 165 (25.4) | 59 (24.7) | 36 (26.9) | 163 (22.5) |  |  |
| **4+** | 225 (34.6) | 84 (35.1) | 46 (34.3) | 160 (22.1) |  |  |
| **Codetection, any positivity** | | | | | | |
| **Single bacterium** | 12 (1.8) | 7 (2.9) | 5 (3.7) | 35 (4.8) | <.0001 | <.0001 |
| **2 or more bacteria (no viruses present)** | 39 (6.0) | 13 (5.4) | 8 (6.0) | 103 (14.2) |  |  |
| **Single virus** | 26 (4.0) | 11 (4.6) | 8 (6.0) | 13 (1.8) |  |  |
| **2 or more viruses (no bacteria present)** | 31 (4.8) | 12 (5.0) | 3 (2.2) | 8 (1.1) |  |  |
| **Bacterial-Viral organisms** | 538 (82.8) | 193 (80.8) | 110 (82.1) | 545 (75.3) |  |  |
| **Codetection, above threshold^b^** | | | | | | |
| **Single bacterium** | 36 (5.5) | 10 (4.2) | 6 (4.5) | 122 (16.9) | <.0001 | <.0001 |
| **2 or more bacteria (no viruses present)** | 45 (6.9) | 19 (7.9) | 13 (9.7) | 86 (11.9) |  |  |
| **Single virus** | 49 (7.5) | 16 (6.7) | 11 (8.2) | 39 (5.4) |  |  |
| **2 or more viruses (no bacteria present)** | 52 (8.0) | 21 (8.8) | 7 (5.2) | 18 (2.5) |  |  |
| **Bacterial-Viral organisms** | 451 (69.4) | 163 (68.2) | 92 (68.7) | 401 (55.4) |  |  |

^a^ Restricted to participants with NP/OP PCR results available.

^b^CXR+ cases: finding of alveolar consolidation, other infiltrate or both on chest radiograph.

^c^Odds ratios adjusted for age (months) and all other pathogens detected on NP/OP PCR. P-values are from logistic regression adjusted for site and age in months.

^d^ Threshold defined using NP/OP PCR density for 4 pathogens: *P. jirovecii*, 4 log10 copies/mL; *H. influenzae*, 5.9 log10 copies/mL; CMV, 4.9 log10 copies/mL; *S. pneumoniae*, 6.9 log10 copies/mL.
