# Supplementary material for: The Etiology of Childhood Pneumonia in Mali: Findings From the Pneumonia Etiology Research for Child Health (PERCH) Study
Source: Pediatr Infect Dis J. 2021 Aug 25;40(9):S18–28. doi: 10.1097/INF.0000000000002767 (PMC8448406; doi:10.1097/INF.0000000000002767)
Supplement: Supplementary file 5 [file inf-40-s18-s005.docx]

**Supplemental Digital Content 5, Table. PERCH NPPCR Results by Age Group, Mali HIV-Uninfected Cases and Controls**

|  | **1-5 months** | | | **6-11 months** | | | **12-23 months** | | | **24-59 months** | | |
| --- | --- | --- | --- | --- | --- | --- | --- | --- | --- | --- | --- | --- |
|  | All Cases | CXR+ Cases^a^ | Controls | All Cases | CXR+ Cases^a^ | Controls | All Cases | CXR+ Cases^a^ | Controls | All Cases | CXR+ Cases^a^ | Controls |
|  | N=299 | N=98 | N=247 | N=148 | N=59 | N=188 | N=137 | N=61 | N=165 | N=69 | N=23 | N=125 |
|  | n (col %) | n (col %) | n (col %) | n (col %) | n (col %) | n (col %) | n (col %) | n (col %) | n (col %) | n (col %) | n (col %) | n (col %) |
| RSV | 123 (41.1) | 36 (36.7) | 10 (4.1) | 19 (12.8) | 7 (11.9) | 6 (3.2) | 16 (11.7) | 8 (13.1) | 5 (3.0) | 6 (8.7) | 3 (13.0) | 7 (5.6) |
| Boca | 24 (8.0) | 9 (9.2) | 19 (7.7) | 31 (21.0) | 10 (17.0) | 30 (16.0) | 23 (16.8) | 11 (18.0) | 23 (13.9) | 13 (18.8) | 7 (30.4) | 10 (8.0) |
| *S. aur* | 74 (24.8) | 26 (26.5) | 57 (23.1) | 27 (18.2) | 16 (27.1) | 8 (4.3) | 20 (14.6) | 9 (14.8) | 8 (4.9) | 12 (17.4) | 2 (8.7) | 11 (8.8) |
| Para 3 | 19 (6.4) | 8 (8.2) | 10 (4.0) | 17 (11.5) | 10 (17.0) | 1 (0.5) | 11 (8.0) | 4 (6.6) | 4 (2.4) | 2 (2.9) | 1 (4.4) | 1 (0.8) |
| *H. inf* | 143 (47.8) | 49 (50.0) | 107 (43.3) | 87 (58.8) | 36 (61.0) | 118 (62.8) | 85 (62.0) | 40 (65.6) | 112 (67.9) | 42 (60.9) | 13 (56.5) | 81 (64.8) |
| B | 7 (2.3) | 3 (3.1) | 6 (2.4) | 4 (2.7) | 1 (1.7) | 3 (1.6) | 3 (2.2) | 3 (4.9) | 5 (3.0) | 1 (1.5) | 0(0) | 0 (0) |
| Not B | 136 (45.5) | 46 (46.9) | 101 (40.9) | 83 (56.1) | 35 (59.3) | 115 (61.2) | 82 (59.9) | 37 (60.7) | 107 (64.9) | 41 (59.4) | 13 (56.5) | 81 (64.8) |
| CMV | 126 (42.1) | 42 (42.9) | 115 (46.6) | 112 (75.7) | 42 (71.2) | 123 (65.4) | 96 (70.1) | 43 (70.5) | 122 (73.9) | 46 (66.7) | 16 (69.6) | 53 (42.4) |

^a^ CXR+ defined as consolidation and/or other infiltrate on chest radiograph.

RSV, Respiratory syncytial virus A/B; Boca, Human bocavirus; S. aur, *Staphylococcus aureus*; Para 3, Parainfluenza virus type 3; H. inf, *Haemophilus influenzae*; CMV, cytomegalovirus
