# Supplementary material for: The Etiology of Childhood Pneumonia in Mali: Findings From the Pneumonia Etiology Research for Child Health (PERCH) Study
Source: Pediatr Infect Dis J. 2021 Aug 25;40(9):S18–28. doi: 10.1097/INF.0000000000002767 (PMC8448406; doi:10.1097/INF.0000000000002767)
Supplement: Supplementary file 6 [file inf-40-s18-s006.docx]

Supplemental Digital Content 6, Figure. Etiology of CXR+, HIV-uninfected Severe and Very Severe Pneumonia by Age Group

CXR+ defined as consolidation and/or other infiltrate on chest radiograph.

<1 yr defined as 28 days to 11 months; ≥1 yr defined as 12-59 months.

Adeno, Adenovirus; B. pert, *Bordetella pertussis*; Boca, Human bocavirus; C. pneu, *Chlamydophila pneumoniae*; Cand sp, Candida species; CMV, cytomegalovirus; Entrb, Enterobacteriaceae; Flu, influenza virus A, B and C; H. inf, *Haemophilus influenzae*; HCoV, Coronavirus; HMPV, Human metapneumovirus A/B; Legio, Legionella species; M. cat, *Moraxella catarrhalis*; M. pneu, *Mycoplasma pneumoniae*; Mtb, *Mycobacterium tuberculosis*; NFGNR, Nonfermentative gram-negative rods; N. men, *Neisseria meningitidis*; NoS, Not Otherwise Specified (i.e., pathogens not tested for); P. jirov, *Pneumocystis jirovecii*; Para, Parainfluenza virus types 1, 2, 3 and 4; PCV, pneumococcal conjugate vaccine; PV/EV, Parechovirus/Enterovirus; Rhino, Human rhinovirus; RSV, Respiratory syncytial virus A/B; S. aur, *Staphylococcus aureus*; S. pneu, *Streptococcus pneumoniae*; Salm sp, *Salmonella* spp.; VPD, vaccine preventable disease.

Other Strep includes *Streptococcus pyogenes* and *Enterococcus faecium*. NFGNR includes *Acinetobacter* spp. and *Pseudomonas* spp. *Enterobacteriaceae* includes *E. coli*, *Enterobacter* spp., and *Klebsiella* spp., excluding mixed gram-negative rods. Vaccine preventable disease includes *S. pneumoniae* PCV13 type, *H. influenzae* type b, and *B. pertussis*.

Bacterial summary excludes Mtb.

Pathogens estimated at the subspecies level are presented grouped and disaggregated (Parainfluenza virus type 1, 2, 3 and 4; *S. pneumoniae* PCV13 and *S. pneumoniae* non-PCV13 types; *H. influenzae* type b and *H. influenzae* non-b; influenza A, B, and C).

Description of symbols: Line represents the 95% credible interval. The size of the symbol is scaled based on the ratio of the estimated etiologic fraction to its standard error. Of two identical etiologic fraction estimates, the estimate associated with a larger symbol is more informed by the data than the priors.
