# Supplementary material for: The Etiology of Childhood Pneumonia in Mali: Findings From the Pneumonia Etiology Research for Child Health (PERCH) Study
Source: Pediatr Infect Dis J. 2021 Aug 25;40(9):S18–28. doi: 10.1097/INF.0000000000002767 (PMC8448406; doi:10.1097/INF.0000000000002767)
Supplement: Supplementary file 9 [file inf-40-s18-s009.docx]

Supplemental Digital Content 9, Table. Laboratory Results by Vital Status (CXR+/HIV- cases)

|  | **Cases who survived through 30 days post admission (N=202)^a^**  **n (%)** | **Cases who died in the hospital or within 30 days of admission (N=31)^a^**  **n (%)** | **p-value^b^** |
| --- | --- | --- | --- |
| **Blood culture** |  |  |  |
| **Any non-contaminant organism** | 11 (5.5) | 1 (3.2) | 0.603 |
| ***Streptococcus pneumoniae*** | 6 (3.0) | 0 (0) | 0.331 |
| ***S. pneumoniae*, vaccine type (PCV13)** | 2 (1.0) | 0 (0) | 0.578 |
| ***S. pneumoniae*, non-vaccine type (PCV13)** | 4 (2.0) | 0 (0) | 0.429 |
| ***Hemophilus influenzae*** | 4 (2.0) | 1 (3.2) | 0.656 |
| ***H. influenzae* type b** | 3 (1.5) | 1 (3.2) | 0.487 |
| ***H. influenzae* non-type b** | 1 (0.5) | 0 (0) | 0.695 |
| **Non-typhoidal *Salmonella*** | 1 (0.5) | 0 (0) | 0.695 |
| **Contaminants^b^** | 13 (6.4) | 3 (9.7) | 0.506 |
| **Induced Sputum / Gastric Aspirate** |  |  |  |
| ***Mycobacterium tuberculosis^c^*** | 1 (0.5) | 0 (0) | 0.726 |
| **Pleural Fluid culture** |  |  |  |
| *Staphylococcus aureus* | 3 (1.5) | 0 (0) | 0.495 |
| *Escherichia coli* | 1 (0.5) | 0 (0) | 0.695 |
| Group F streptococcus | 1 (0.5) | 0 (0) | 0.695 |
| **Pleural Fluid PCR** |  |  |  |
| *S. pneumoniae* | 2 (1.0) | 0 (0) | 0.578 |
| *H. influenzae non-type b* | 1 (0.5) | 0 (0) | 0.695 |
| **Nasopharyngeal/oropharyngeal PCR - Bacteria** |  |  |  |
| Any Pathogen | 199 (99.5) | 30 (96.8) | 0.127 |
| Any bacteria | 182 (91.0) | 26 (83.9) | 0.217 |
| Any bacteria, with thresholds applied for *S. pneumoniae and H. influenzae* | 165 (81.7) | 24 (77.4) | 0.572 |
| *S. pneumoniae* | 145 (72.5) | 19 (61.3) | 0.201 |
| >6.9 log10 copies/ml | 53 (26.5) | 7 (22.6) | 0.643 |
| PCV13-type with >6.9 log_10_ copies/ml | 26 (13.0) | 4 (12.9) | 0.988 |
| Non PCV13-type with >6.9 log_10_ copies/ml | 26 (20.3) | 3 (14.3) | 0.518 |
| *H. influenzae* | 121 (60.5) | 13 (41.9) | 0.051 |
| >=5.9 log10 copies/ml | 75 (37.5) | 6 (19.4) | **0.049** |
| *H. influenzae* not type b | 117 (58.5) | 11 (35.5) | **0.016** |
| *H. influenza*e not type b >=5.9 log10 copies/ml | 71 (35.5) | 5 (16.1) | **0.033** |
| *H. influenzae* type b | 4 (2.0) | 2 (6.5) | 0.147 |
| *H. influenzae* type b >=5.9 log10 copies/ml | 4 (2.0) | 1 (3.2) | 0.663 |
| *S. aureus* | 42 (21.0) | 11 (35.5) | 0.074 |
| *C. pneumoniae* | 4 (2.0) | 0 (0) | 0.427 |
| *M. catarrhalis* | 131(65.5) | 15 (48.4) | 0.066 |
| *M. pneumoniae* | 3 (1.5) | 0 (0) | 0.492 |
| Salmonella species | 4 (2.0) | 0 (0) | 0.427 |
| Legionella | 0 (0) | 0 (0) | n/a |
| *B. pertussis* | 2 (1.0) | 1 (3.2) | 0.308 |
| **Nasopharyngeal/oropharyngeal PCR - Viruses** |  |  |  |
| Any virus | 183 (91.5) | 26 (83.9) | 0.178 |
| Any virus, with thresholds applied for CMV | 168 (83.2) | 25 (80.7) | 0.729 |
| Adenovirus | 22 (11.0) | 6 (19.4) | 0.185 |
| CMV | 121(60.5) | 18 (58.1) | 0.797 |
| >4.9 log10 copies/ml | 48 (24.0) | 11(35.5) | 0.172 |
| Coronavirus OC43 | 2 (1.0) | 1 (3.2) | 0.308 |
| Coronavirus NL63 | 6 (3.0) | 1 (3.2) | 0.946 |
| Coronavirus HKU1 | 9 (4.5) | 1 (3.2) | 0.746 |
| Coronavirus 229E | 3 (1.5) | 0 (0) | 0.492 |
| HBOV | 31 (15.5) | 5 (16.1) | 0.928 |
| HMPV A/B | 23 (11.5) | 0 (0) | 0.047 |
| Influenza A | 5 (2.5) | 1 (3.2) | 0.813 |
| Influenza B | 1 (0.5) | 0 (0) | 0.693 |
| Influenza C | 1 (0.5) | 0 (0) | 0.693 |
| Parainfluenza 1 | 3 (1.5) | 1 (3.2) | 0.493 |
| Parainfluenza 2 | 3 (1.5) | 2 (6.5) | 0.078 |
| Parainfluenza 3 | 19 (9.5) | 3 (9.7) | 0.975 |
| Parainfluenza 4 | 3 (1.5) | 0 (0) | 0.492 |
| PV/EV | 20 (10.0) | 4 (12.9) | 0.622 |
| Rhinovirus | 29 (14.5) | 4 (12.9) | 0.813 |
| RSV | 47 (23.5) | 3 (9.7) | 0.082 |
| **Nasopharyngeal/oropharyngeal PCR - Viruses** |  |  |  |
| *P. jirovecii* | 16 (8.0) | 7 (22.6) | **0.012** |
| >4 log10 copies/ml | 6 (3.0) | 4 (12.9) | **0.012** |

^a^Denominator was those with test available. For blood culture: 202 surviving and 31 fatal cases; for TB testing 196 surviving and 24 fatal cases; for pleural fluid results all participants were used in denominator (202 surviving and 31 fatal cases) because the number with testing available was too small to be informative as a denominator; for nasopharyngeal/oropharyngeal PCR 200 surviving and 31 fatal cases.

^b^ p-values calculated using chi-square test.
