# Supplementary material for: The Etiology of Childhood Pneumonia in Mali: Findings From the Pneumonia Etiology Research for Child Health (PERCH) Study
Source: Pediatr Infect Dis J. 2021 Aug 25;40(9):S18–28. doi: 10.1097/INF.0000000000002767 (PMC8448406; doi:10.1097/INF.0000000000002767)
Supplement: Supplementary file 10 [file inf-40-s18-s010.docx]

Supplemental Digital Content 10, Figure. Etiology by Vital Status among CXR+, HIV-uninfected Cases

Adeno, Adenovirus; B. pert, *Bordetella pertussis*; Boca, Human bocavirus; C. pneu, *Chlamydophila pneumoniae*; Cand sp, Candida species; CMV, cytomegalovirus; Entrb, Enterobacteriaceae; Flu, influenza virus A, B and C; H. inf, *Haemophilus influenzae*; HCoV, Coronavirus; HMPV, Human metapneumovirus A/B; Legio, Legionella species; M. cat, *Moraxella catarrhalis*; M. pneu, *Mycoplasma pneumoniae*; Mtb, *Mycobacterium tuberculosis*; NFGNR, Nonfermentative gram-negative rods; N. men, *Neisseria meningitidis*; NoS, Not Otherwise Specified (i.e., pathogens not tested for); P. jirov, *Pneumocystis jirovecii*; Para, Parainfluenza virus types 1, 2, 3 and 4; PCV, pneumococcal conjugate vaccine; PV/EV, Parechovirus/Enterovirus; Rhino, Human rhinovirus; RSV, Respiratory syncytial virus A/B; S. aur, *Staphylococcus aureus*; S. pneu, *Streptococcus pneumoniae*; Salm sp, *Salmonella* spp.; VPD, vaccine preventable disease.

Other Strep includes *Streptococcus pyogenes* and *Enterococcus faecium*. NFGNR includes *Acinetobacter* spp. and *Pseudomonas* spp. *Enterobacteriaceae* includes *E. coli*, *Enterobacter* spp., and *Klebsiella* spp., excluding mixed gram-negative rods. Vaccine preventable disease includes *S. pneumoniae* PCV13 type, *H. influenzae* type b, and *B. pertussis*.

Died=Died in the hospital or within 30 days of hospital admission; Alive=Followed up within 21-90 days of hospital admission and found to be alive.

CXR+ defined as consolidation and/or other infiltrate on chest radiograph. Bacterial summary excludes Mtb.

Pathogens estimated at the subspecies level are presented grouped and disaggregated (Parainfluenza virus type 1, 2, 3 and 4; *S. pneumoniae* PCV 13 and *S. pneumoniae* non PCV 13 types; *H. influenzae* type b and *H. influenzae* non b; influenza A, B, and C).

Description of symbols: Color of symbols: Red=bacteria; Blue=viruses; Gray=other.

Line represents the 95% credible interval. The size of the symbol is scaled based on the ratio of the estimated etiologic fraction to its standard error. Of two identical etiologic fraction estimates, the estimate associated with a larger symbol is more informed by the data than the priors.
