# Supplementary material for: The Etiology of Childhood Pneumonia in Mali: Findings From the Pneumonia Etiology Research for Child Health (PERCH) Study
Source: Pediatr Infect Dis J. 2021 Aug 25;40(9):S18–28. doi: 10.1097/INF.0000000000002767 (PMC8448406; doi:10.1097/INF.0000000000002767)
Supplement: Supplementary file 11 [file inf-40-s18-s011.docx]

**Supplemental Digital Content 11, Table. Demographic and clinical characteristics of HIV-negative cases, by CXR status**

|  | CXR+ Cases^a^  (N=241) | CXR-Normal Cases  (N=270) | Case with missing CXR  (N=77) | CXR+ Cases vs. CXR-normal Cases | CXR+ Cases vs. CXR-missing Cases |
| --- | --- | --- | --- | --- | --- |
|  | n (%) | n (%) | n (%) | Odds ratio (95% CI)^b^ | Odds ratio (95% CI)^b^ |
| Age |  |  |  |  |  |
| Median age in months (IQR) | 8 (3, 13) | 7 (3, 13) | 7 (3, 16) |  |  |
| 28 days - 5 months | 98 (40.7) | 65 (24.1) | 32 (41.6) | 1.17 (0.65, 2.13) | 1.73 (0.79, 3.81) |
| 6 - 11 months | 59 (24.5) | 52 (19.3) | 18 (23.4) | 1.30 (0.69, 2.13) | 1.85 (0.78, 4.38) |
| 12 - 23 months | 61 (25.3) | 33 (12.2) | 14 (18.2) | 1.68 (0.88, 3.22) | **2.46 (1.00, 6.02)** |
| 24 - 59 months | 23 (9.5) | 115 (42.6) | 13 (16.9) | Ref^c^ | ref |
| Female | 97 (40.2) | 120 (44.4) | 43 (55.8) | 0.91 (0.64, 1.30) | 0.54 (0.32, 0.91) |
| HIV exposure/infection status^d^ |  |  |  |  |  |
| HIV unexposed/uninfected | 158 (65.6) | 183 (67.8) | 50 (64.9) | ref | ref |
| HIV exposed/uninfected | 6 (2.5) | 1 (0.4) | 0 (0.0) | 6.92 (0.82, 58.22) | - |
| Unknown exposure/infection status | 77 (32) | 86 (31.9) | 27 (35.1) | 1.04 (0.71, 1.51) | 0.88 (0.51, 1.52) |
| Pentavalent: fully vaccinated for age^e^ |  |  |  |  |  |
| < 1 year old | 112 (72.7) | 146 (79.4) | 32 (65.3) | 0.68 (0.41, 1.14) | 1.40 (0.70, 2.80) |
| >= 1 year old | 69 (85.2) | 77 (91.7) | 22 (84.6) | 0.57 (0.21, 1.55) | 1.19 (0.34, 4.18) |
| PCV fully vaccinated for age^e^ |  |  |  |  |  |
| < 1 year old | 112 (72.7) | 147 (79.9) | 31 (63.3) | 0.66 (0.40, 1.10) | 1.53 (0.77, 3.04) |
| >= 1 year old | 48 (59.3) | 53 (63.1) | 15 (57.7) | 0.61 (0.30, 1.23) | 0.76 (0.28, 2.02) |
| >1 measles vaccine doses (children > 10 m) | 78 (78.0) | 82 (80.4) | 22 (78.6) | 0.94 (0.47, 1.87) | 1.34 (0.46, 3.93) |
| Mid-upper arm circumference <11.5cm (children ≥ 6m) | 36 (25.2) | 19 (12.7) | 8 (17.8) | **2.26 (1.22, 4.21)** | 1.43 (0.60, 3.40) |
| Weight-for-age z score (WAZ) by WHO standards |  |  |  |  |  |
| ≥ -2 | 127 (52.7) | 185 (68.5) | 36 (55.4) | ref | ref |
| -3 < to < -2 | 43 (17.8) | 54 (20.0) | 12 (18.5) | 1.17 (0.73, 1.84) | 1.19 (0.55, 2.56) |
| < -3 | 71 (29.5) | 31 (11.5) | 17 (26.2) | **3.34 (2.07, 5.39)** | 1.20 (0.61, 2.35) |
| Weight-for-height z score (WHZ) by WHO standards |  |  |  |  |  |
| ≥-2 | 137 (56.9) | 178 (65.9) | 44 (67.7.) | ref | ref |
| -3 < to < -2 | 41 (17.0) | 51 (18.9) | 11 (16.9) | 1.05 (0.66, 1.68) | 1.07 (0.49, 2.34) |
| < -3 | 63 (26.1) | 41 (15.2) | 10 (15.4) | **2.00 (1.27, 3.14)** | 1.58 (0.72, 3.45) |
| Height-for-age z score (HAZ) by WHO standards |  |  |  |  |  |
| ≥ -2 | 177 (73.4) | 234 (86.7) | 46 (70.8) | ref | ref |
| -3 < to < -2 | 34 (14.1) | 24 (8.9) | 7 (10.8) | **1.87 (1.07, 3.27)** | 1.30 (0.52, 3.24) |
| < -3 | 30 (12.5) | 12 (4.4) | 12 (18.5) | **3.30 (1.64, 6.63)** | 0.64 (0. 29, 1.40) |
| Hypoxemia | 133 (55.2) | 95 (35.2) | 45 (58.4) | **2.28 (1.59, 3.25)** | 0.84 (0.50, 1.42) |
| Tachypnea | 205 (85.1) | 225 (83.3) | 69 (89.6) | 1.15 (0.71, 1.86) | 0.70 (0.31, 1.59) |
| Tachycardia | 145 (60.2) | 151 (55.9) | 43 (55.8) | 1.19 (0.84, 1.69) | 1.21 (0.72, 2.04) |
| Very Severe Pneumonia | 96 (39.8) | 115 (57.4) | 65 (84.4) | **0.49 (0.34, 0.70)** | **0.11 (0.06, 0.22)** |
| Danger signs |  |  |  |  |  |
| Head nodding | 58 (24.1) | 79 (29.3) | 35 (45.5) | 0.75 (0.50, 1.13) | **0.35 (0.20, 0.61)** |
| Central cyanosis | 17 (7.1) | 8 (3.0) | 11 (14.3) | **2.48 (1.05, 5.86)** | **0.43 (0.19, 0.97)** |
| Multiple or prolonged seizures | 17 (7.1) | 53 (19.6) | 12 (15.6) | 0.30 (0.17, 0.54) | **0.43 (0.19, 0.95)** |
| Lethargy | 18 (7.5) | 44 (16.3) | 22 (28.6) | 0.40 (0.22, 0.73) | **0.20 (0.10, 0.41)** |
| Unable feed | 32 (13.3) | 45 (16.7) | 33 (42.9) | 0.76 (0.47, 1.25) | **0.20 (0.11, 0.36)** |
| Vomiting | 6 (2.5) | 4 (1.5) | 2 (2.6) | 1.69 (0.47, 6.08) | 0.92 (0.18, 4.66) |
| Lung Sounds |  |  |  |  |  |
| Rales | 177 (73.4) | 149 (55.2) | 41 (53.3) | **2.26 (1.56, 3.29)** | 2.39 (1.41, 4.08) |
| Elevated temperature (>38° C) | 218 (90.5) | 232 (85.39) | 68 (88.3) | 1.56 (0.90, 2.70) | 1.28 (0.56, 2.91) |
| Blood culture positive with non- contaminant organism | 12 (5.0) | 9 (3.3) | 11 (14.3) | 1.52 (0.63. 3.67) | 1.00 (0.97, 1.03) |
| Leukocytosis | 86 (35.9) | 58 (21.6) | 31 (41.3) | **2.04 (1.38, 3.03)** | 0.81 (0.48, 1.38) |
| CRP >= 40 mg/L | 89 (43.0) | 59 (25.0) | 30 (41.7) | **2.45 (1.60, 3.76)** | 1.13 (0.64, 2.00) |
| Hemoglobin |  |  |  |  |  |
| 0 - 7.5 g/dL | 44 (18.3) | 36 (13.4) | 14 (18.7) | 1.31 (0.81, 2.11) | 1.05 (0.54, 2.07) |
| 7.6 - 13.5 g/dL | 190 (79.2) | 223 (82.9) | 61 (81.3) | (ref) | (ref) |
| >13.5 g/dL | 6 (2.5) | 10 (3.7) | 0 (0.0) | 0.77 (0.27, 2.19) | - |
| Duration of illness prior to admission |  |  |  |  |  |
| Median in days (IQR) | 5 (3, 7) | 4 (3, 7) | 4 (3, 7) |  |  |
| 0 - 2 days | 17 (7.1) | 41 (15.2) | 13 (16.9) | ref | ref |
| 3 - 5 days | 109 (45.4) | 148 (54.8) | 35 (45.5) | 1.77 (0.96, 3.28) | **2.33 (1.03, 5.29)** |
| >5 days | 114 (47.5) | 81 (30.0) | 29 (37.7) | **3.38 (1.80, 6.37)** | **2.95 (1.28, 6.78)** |
| Duration of hospitalization |  |  |  |  |  |
| Median in days (IQR) | 7 (4, 11) | 5 (3, 7) | 1 (0,7) |  |  |
| 0 - 2 days | 10 (4.2) | 18 (6.7) | 42 (54.6) | ref | ref |
| 3 - 5 days | 83 (34.4) | 144 (53.3) | 12 (15.6) | 1.03 (0.46, 2.35) | **29.16 (11.63, 73.11)** |
| >5 days | 148 (61.4) | 108 (40.0) | 23 (29.9) | **2.46 (1.09, 5.55)** | **26.87 (11.85, 60.91)** |
| Died |  |  |  |  |  |
| Any time within 30 days of admission^f^ | 31 (13.3) | 24 (8.9) | 42 (56.8) | 1.52 (0.86, 2.67) | **0.11 (0.06, 0.21)** |
| In hospital | 25 (10.4) | 20 (7.4) | 42 (54.6) | 1.44 (0.78, 2.67) | **0.10 (0.05, 0.18)** |
| After discharge but within 30 days of admission | 6 (2.9) | 4 (1.7) | 0 (0.0) | 1.77 (0.49, 6.38) | - |

^a^ CXR+ cases: finding of alveolar consolidation, other infiltrate, or both on chest radiograph.

^b^ Odds ratios calculated using logistic regression adjusting for age in months (except of age category where no age adjustment was performed).

^c^ Reference category.

^d^ Positive ELISA test or 2) reported maternal history of HIV+ during pregnancy if child is <7 months without a negative ELISA result or 3) reported maternal history of HIV+ during pregnancy if child is ≥ 7 months regardless of ELISA results

^e^ For children < 1 year, defined as received at least one dose and up-to-date for age based on the child's age at enrolment, doses received, and country schedule (allowing 4-week window each for dose). For children > 1 year, defined as 3+ doses.

^g^Among those with vital status available at 30 days following admission: N=233 for CXR+; N=262 for CXR normal; N=64 for CXR missing.
