# Supplementary material for: The Etiology of Childhood Pneumonia in Mali: Findings From the Pneumonia Etiology Research for Child Health (PERCH) Study
Source: Pediatr Infect Dis J. 2021 Aug 25;40(9):S18–28. doi: 10.1097/INF.0000000000002767 (PMC8448406; doi:10.1097/INF.0000000000002767)
Supplement: Supplementary file 12 [file inf-40-s18-s012.docx]

**Supplemental Digital Content 12, Table. Mortality by HIV and Nutritional Status, CXR+ Cases**

|  | **n/N (%) fatal^1^** | **n/N (%) fatal** | **n/N (%) fatal** |
| --- | --- | --- | --- |
|  | **HIV-negative and unexposed** | **HIV positive or exposed** | **HIV status unknown** |
| Weight-for-age z score (WAZ) by WHO standards |  |  |  |
| ≥-2 | 3/84 (3.6) | 0/5 (0.0) | 3/37 (8.1) |
| -3 to < -2 | 4/27 (14.8) | 0/1 (0.0) | 2/15 (13.3) |
| <-3 | 13/43 (30.2) | 4/10 (40.0) | 5/22 (22.7) |
| Weight-for-height z score (WHZ) by WHO standards |  |  |  |
| ≥-2 | 6/94 (6.4) | 0/6 (0.0) | 3/35 (8.6) |
| -3 to < -2 | 3/24 (12.5) | 0/2 (0.0) | 2/17 (11.8) |
| <-3 | 11/36 (30.6) | 4/8 (50.0) | 5/22 (22.7) |
| Height-for-age z score (HAZ) by WHO standards < - 3 |  |  |  |
| ≥-2 | 13/108 (12.0) | 1/7 (14.3) | 7/62 (11.3) |
| -3 to < -2 | 2/23 (8.7) | 0/2 (0.0) | 2/8 (25.0) |
| <-3 | 5/23(21.7) | 3/7 (42.9) | 1/4 (25.0) |

^1^Cases who died in the hospital or within 30 days of admission- restricted to those with mortality status known at 30 days post admission)
